# Vaccine Potential and Diversity of the Putative Cell Binding Factor (CBF, NMB0345/NEIS1825) Protein of *Neisseria meningitidis*

**DOI:** 10.1371/journal.pone.0160403

**Published:** 2016-08-09

**Authors:** María Victoria Humbert, Miao-Chiu Hung, Renee Phillips, Charlene Akoto, Alison Hill, Wei-Ming Tan, John Edward Heckels, Myron Christodoulides

**Affiliations:** Neisseria Research, Molecular Microbiology, Academic Unit of Clinical and Experimental Sciences, Sir Henry Wellcome Laboratories, University of Southampton Faculty of Medicine, Southampton, United Kingdom; University of Cambridge, UNITED KINGDOM

## Abstract

The *cbf* gene from *Neisseria meningitidis* strain MC58 encoding the putative Cell Binding Factor (CBF, NMB0345/NEIS1825) protein was cloned into the pRSETA system and a ~36-kDa recombinant (r)CBF protein expressed in *Escherichia coli* and purified by metal affinity chromatography. High titres of rCBF antibodies were induced in mice following immunization with rCBF-saline, rCBF-Al(OH)_3_, rCBF-Liposomes or rCBF-Zwittergent (Zw) 3–14 micelles, both with and without incorporated monophosphoryl lipid A (MPLA) adjuvant. Anti-rCBF sera reacted in western blots of meningococcal lysates with a single protein band of molecular mass ~29.5 kDa, indicative of mature CBF protein, but did not react with a lysate of a Δ*nmb0345* mutant (CBF^-^), demonstrating specificity of the murine immune responses. CBF protein was produced by all strains of meningococci studied thus far and the protein was present on the surface of MC58 (CBF^+^) bacteria, but absent on Δ*nmb0345* mutant (CBF^-^) bacteria, as judged by FACS reactivity of anti-rCBF sera. Analysis of the NEIS1825 amino acid sequences from 6644 *N*. *meningitidis* isolates with defined Alleles in the pubmlst.org/*Neisseria* database showed that there were 141 ST types represented and there were 136 different allelic loci encoding 49 non-redundant protein sequences. Only 6/6644 (<0.1%) of *N*. *meningitidis* isolates lacked the *nmb0345* gene. Amongst serogroup B isolates worldwide, ~68% and ~20% expressed CBF encoded by Allele 1 and 18 respectively, with the proteins sharing >99% amino acid identity. Murine antisera to rCBF in Zw 3–14 micelles + MPLA induced significant serum bactericidal activity (SBA) against homologous Allele 1 and heterologous Allele 18 strains, using both baby rabbit serum complement and human serum complement (h)SBA assays, but did not kill strains expressing heterologous protein encoded by Alelle 2 or 3. Furthermore, variable bactericidal activity was induced by murine antisera against different meningococcal strains in the hSBA assay, which may correlate with variable surface exposure of CBF. Regardless, the attributes of amino acid sequence conservation and protein expression amongst different strains and the ability to induce cross-strain bactericidal antibodies indicates that rCBF could be a potential meningococcal vaccine antigen and merits further testing.

## Introduction

Vaccination is the most effective prophylaxis for sepsis and meningitis caused by *Neisseria meningitidis* (meningococcus). Capsular polysaccharide (CPS)-conjugate vaccines protect against infections caused by serogroup A (MenA), C (MenC), Y (MenY) and W (MenW) meningococci [1, 2], but this strategy is not effective for serogroup B (MenB) meningococcal CPS [3]. Alternatively, sub-capsular MenB outer membrane (OM) vesicle vaccines have successfully controlled clonal epidemics worldwide, but they provide no significant cross-strain protection [4]. *In silico* reverse vaccinology and proteomic technologies have been used to develop the Bexsero^®^ [5] and Trumenba^®^ [6–8] MenB vaccines, respectively. Bexsero^®^ was licensed in 2013 by the European Medicines Agency for use in the European Union and recommended for infant use in the UK [9, 10] and has been used also to control outbreaks of MenB infection at two US universities [11]. Trumenba^®^ has been recommended for use in adolescents [12, 13]. Strain coverage is a potential concern with these new MenB vaccines, with varying estimates for Bexsero^®^ in several European countries (73–89%), North America (66–88%), Australia (76%) and Brazil (81%) [14–16]. A study of predictive coverage by Bexsero^®^ in the Gipuzkoa region of Northern Spain during 2008–2013 estimated rates of 61.8% and 50% respectively for MenB and non-MenB meningococci, which fell to 45% during 2012–2013 [17]. Protective bactericidal titers induced by Trumenba^®^ for toddlers and adolescents-young adults, ranged from 44–100% and from 68–98%, respectively, against MenB strains expressing heterologous vaccine proteins [7].

The meningococcal OM contains a large number of potential surface-exposed protein vaccine candidates. Immuno-proteomic study of serum obtained before and after colonisation or infection with meningococci [18] demonstrated that increased reactivity with specific OM proteins correlated with increased serum bactericidal activity (SBA), the generally accepted correlate of protection in humans [19]. One of these proteins was a putative Cell Binding Factor (CBF; NMB0345 in MenB strain MC58; NMA2142 in MenA strain Z4970; NEIS1825, pubmlst.org/*Neisseria*). CBF has homology to a major antigenic protein from *Campylobacter jejuni*, Cj0596 (PEB4, Cell Binding Factor 2, CBF2), which has been shown to be important for bacterial adherence to murine INT407 cells, for colonization of mouse intestinal epithelium and for biofilm formation [20]. Using an infant rat model of meningococcal infection, Sun *et al*. [21] identified NMB0345/CBF as one of 73 genes of MenB strain C311+ essential for meningococcal septicaemia. NMB0345 has been reported to contribute towards meningococcal adherence to epithelial cells [22] and potentially to colonization [23]. The *nmb0345* gene was also up-regulated in the presence of human haemoglobin [24] and was implicated in survival in human blood [22]. To our knowledge, the vaccine potential of CBF has not been reported and in the current study we provide evidence that CBF is a highly conserved, expressed and surface accessible protein capable of inducing bactericidal antibodies.

## Materials and Methods

### Bacteria, growth conditions and preparation of OM

*Neisseria meningitidis* strain MC58 (B: 15:P1.7,16b) and 12 other meningococcal strains of different CPS serogroups, PorB protein serotypes, and PorA protein serosubtypes, originally isolated from patients orhh colonized individuals, have been described previously [18, 25]. *N*. *meningitidis* strains M15 240010 (W: P1.5,2: F1-1: ST-11 (cc11)), M15 240190 (Y: P1.5,2: F5-58: ST-11 (cc11)), M15 240016 (B: P1.12–1,16: F1-5: ST-485 (cc41/44)), M15 240120 (B: P1.22,14: F3-6: ST-2100 (cc213)) and M15 240106 (B: P1.7–1,1: F1-5: ST-41 (cc41/44)) were obtained from the Public Health England Meningococcal Reference Unit, Manchester. MC58 *Δnmb0345* mutant strain was obtained from K. Wooldridge, University of Nottingham. Meningococci were grown on supplemented GC agar plates incubated at 37°C with an atmosphere of 5% (vol/vol) CO_2_ [18]. *E*. *coli* DH5α (cloning) and BL21(DE3) pLysS (protein expression) strains (Invitrogen, UK) were grown on Luria-Bertani (LB) agar, LB broth or SOB with or without addition of selective antibiotics. Whole meningococcal suspensions were prepared in water and OM was prepared by extraction of whole bacteria with 0.2 M lithium acetate buffer, pH 5.8 [26] and differential ultracentrifugation [27].

### Cloning and expression of the *cbf* gene in *E*. *coli*

Genomic DNA of MC58 was extracted by alkaline lysis [25] and used as the PCR template. The *cbf* gene sequence encoding the entire coding sequence for the NMB0345 protein (867 bp, NCBI), including the signal leader peptide and the following lipidation site (Cys21) was amplified using forward primer NMB0345F (5’-ggctatctcgagatgaaagcaaaaatcctgac-3’), reverse primer NMB0345R (5’-ggctataagcttctattattttgcaggtttgatgt -3’) and 2× Phusion PCR master mix (ThermoScientific, UK) under the following PCR conditions: initial denaturation (98°C, 30 s), 30 cycles of denaturation (98°C, 10 s), annealing (57°C, 30 s) and extension (72°C, 26 s) and a final extension (72°C for 5 min). The restriction site sequences for XhoI and HindIII enzymes are ctcgag and aagctt, respectively. The method for gene cloning into the pRSETA system with an N-terminus HIS-tag was described previously [25]. Recombinant plasmids carrying the *cbf* gene, which was confirmed by sequence analysis, were transformed into *E*. *coli* BL21(DE3) pLysS and protein expression was induced by addition of isopropyl-β-d-thiogalactopyranoside (1 mM final concentration), followed by bacterial growth for 4 h.

### Purification of rCBF

rCBF protein was purified using nickel-nitrilotriacetic acid metal-affinity chromatography under denaturing conditions (QIAexpressionist; Qiagen, UK). Bound protein was eluted with 100 mM NaH_2_PO_4_ buffer, 10 mM Tris, 8 M urea buffer, pH 4.5. Samples obtained during purification were analyzed using 12% (wt/vol) SDS-PAGE electrophoresis (Mini-protean system, Bio-Rad, UK), then pooled and precipitated using 100% (vol/vol) ethanol overnight. The protein pellet was solubilised in phosphate buffered saline (PBS, pH7.4) containing 0.5% (wt/vol) sodium dodecyl sulphate (SDS) and the protein concentration was determined using the bicinchoninic acid assay (ThermoScientific, UK). Purity of rCBF protein was assessed by SDS-PAGE and western blot reactivity with rabbit anti-rCBF antiserum (1/400 dilution) and mouse anti-HIS tag antibody (Invitrogen 372900, 1/500).

### Sequencing the *cbf* gene

The *cbf* gene of selected meningococcal strains was sequenced commercially (Source BioScience, Oxford, UK) using primers s0345F (5’-gtcaaatcgctgctcaaaga-3’) and s0345R (5’- caccagcgtatccaccaaag-3’). Additional meningococcal *cbf* gene (NEIS1825) sequences were obtained from http://pubmlst.org/neisseria/ and sequence alignments generated using Clustal (http://www.ebi.ac.uk/Tools/msa/clustalo/) and a dendrogram assembled with Jalview 2.8 (www.jalview.org).

### Immunization of animals

BALB/c mice (H-2^d^ haplotype) and New Zealand White (NZW) rabbits were housed under standard conditions of temperature and humidity with a 12h lighting cycle and with food and water available *ad libitum*. BALB/c mice were bred within the animal facilities of the university and NZW rabbits were purchased from Harlan, UK (now Envigo). Groups of five male BALB/c mice of approximate equal sizes and weights (6–7 weeks of age) were immunized intraperitoneally with rCBF-saline, rCBF-Al(OH)_3_, rCBF-liposomes ± MPLA, and rCBF-Zwittergent (Zw) 3–14 micelles ± MPLA formulations, prepared using methods described previously [25]. For immunization, the stock protein, solubilized in 0.5% (wt/vol) SDS in PBS was diluted in saline for the preparation of working stocks. The immunization schedule was rCBF (20 μg/mouse) on days 0, 14, and 28. Groups of five mice were also sham-injected with the same formulations without rCBF and one group was kept for normal serum. Generation of murine antisera to MC58 OM has been described previously [25]. Mice were terminally bled by cardiac puncture under anesthesia on day 42.

Female NZW rabbits (*n* = 2) were immunized subcutaneously with rCBF (50 μg/dose) emulsified in Freund’s complete adjuvant (dose I) and Freund’s incomplete adjuvant (doses II-IV) at 14-day intervals. Rabbits were terminally bled from the middle ear vein followed by cardiac puncture under anesthesia, 14 days after the last dose. All sera were stored at −20°C until required. This study complied with the animal experimentation guidelines of the Home Office, with approval granted under the Animals (Scientific Procedures) Act, 1986) and was approved by the Animal Welfare and Ethics Review Board (AWERB) at the authors’ institution (University of Southampton). Animal health and welfare was assessed daily by qualified animal technicians and no animals suffered significant adverse effects.

### Characterization of biological and functional properties of antibodies to rCBF

#### Enzyme-linked immunosorbent assay (ELISA)

Individual sera were reacted with rCBF and homologous OM in ELISA, as described previously [28]. Sera were also reacted against whole MC58 meningococcal cells: briefly, from an overnight agar plate culture of meningococci, bacterial growth was suspended in PBS and the concentration in colony forming units (CFU) was determined by viable counting. ELISA wells were coated with 100μl of carbonate coating buffer, pH9.6 containing 1×10^7^ CFU and subsequent steps for the whole bacterial cell ELISA were identical to the rCBF and OM ELISA. Absorbance was measured at λ450 nm after 10 min of incubation with enzyme substrate, and the ELISA titer, extrapolated from the linear portion of the serum titration curve, was taken as the reciprocal dilution which gave an increase in absorbance of 0.1 U after 10 min. An independent t-Test was used to compare differences between mean values for ELISA data, with p values <0.05 considered significant.

#### SDS-PAGE and western immunoblotting

OM or whole-cell lysate preparations (10 μg/lane) were separated on SDS-PAGE, transferred to nitrocellulose using semi-dry blotting and immunological reactivity detected as described previously [28].

#### Fluorescence-activated cell sorter (FACS) analysis

Methods for sample preparation, antibody binding to whole bacteria, detection with FITC-conjugated species-specific IgG reagents and analyses on a FACSAria flow cytometer (BD Biosciences), have all been described previously [29]. A one sample t-Test was used to compare the mean fluorescence values for antisera raised to rCBF and corresponding sham-immunized sera when tested against parent MC58 (CBF^+^) bacteria and the MC58Δ*nmb0345* (CBF^-^) knock-out strain, with p values <0.05 considered significant. A one-sample t-Test was also used to compare the mean fluorescence values for rabbit antiserum to rCBF and the corresponding pre-immune serum, tested against MC58 wild-type and knock-out strains.

#### Complement-activated killing of meningococci

The bactericidal activities of pooled antisera were determined with 5% (v/v) baby rabbit serum complement (BRC) (AbD Serotec, UK) and 25% (v/v) Ig-depleted human serum complement (HC) as described previously [28, 30]. Quality control of human complement source was determined as described previously [30]. Complement-dependent bactericidal activity was determined from the numbers of bacteria surviving in the presence of serum and complement compared to the numbers surviving with complement but without test serum. Sera that showed bactericidal activity (>50%) in two or more dilutions were considered positive and statistical significance was accepted with p values <0.05 as determined with a two-sample t-Test, comparing the CFU survivors in the presence of serum and complement with the CFU survivors in the presence of complement alone (no serum).

## Results

### Bioinformatic analysis

**Fig 1:**
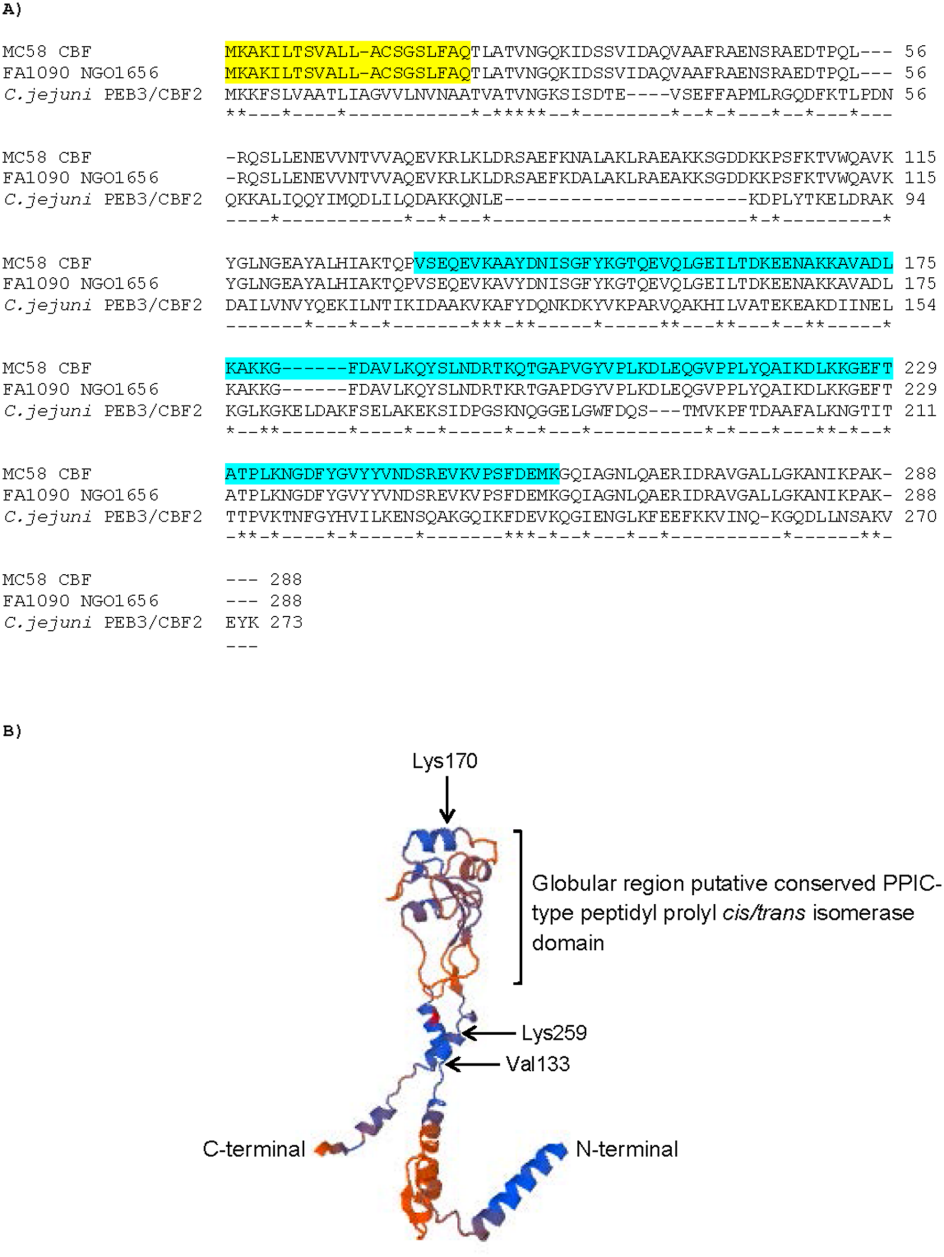
A. Amino acid sequence alignment of NMB0345 (NEIS1825, Cell Binding Factor, CBF) from *N*. *meningitidis* MC58, *N*.*gonorrhoeae* FA1090 and the *Campylobacter jejuni* PEB3/CBF2 protein. * denotes identical amino acids between all three proteins. Yellow-shaded amino acid sequences denote the putative signal leader peptides. The blue-shaded amino acid sequence denotes the presence of a putative conserved PPIC-type peptidyl prolyl *cis/trans* isomerase domain (interval 133–259) belonging to the Rotamase_2 superfamily. **B) Putative structure of meningococcal CBF based on *C*.*jejuni* CBF**. The structure of meningococcal CBF was putatively modelled in SWISS-model, using as a template the crystallized structure of 3rfw.1.A protein (PEB4), which is the most closely related structure to *C*. *jejuni* PEB3/CBF2 that we have available in Protein Data Bank.

Full-length CBF (NMB0345) is a protein of 288 amino acids and is predicted to be a lipoprotein with a cleavage site between amino acids 20–21, mediated by type I signal peptidase (http://www.cbs.dtu.dk/services/LipoP/) (Fig 1A). Full-length CBF has a predicted molecular mass (*Mr*) of 31455 daltons and an average isoelectric point (IP) of 9.636, whilst the mature protein has a predicted *Mr* of 29448 daltons and an average IP of 9.555 (Kozlowski LP. 2007–2013 Isoelectric Point Calculator. http://isoelectric.ovh.org). Using the ProtParam tool (http://web.expasy.org/protparam/), estimation of CBF hydropathy (as a Grand Average Hydropathy (GRAVY) score) from the amino acid sequence was -0.436, with 36 negatively charged residues (Asp, Glu) and 44 positively charged residues (Arg, Lys). The negative score denoted greater hydrophilicity of the protein. Although PSORTb (version 3.0.2. http://www.psort.org/psortb/) could not predict protein location (unknown), the fact that CBF is a lipoprotein with a cleavable signal peptide suggest that it is membrane localized. Initially, we used BLAST protein amino acid sequence analysis (NCBI) to investigate the presence of CBF homologues within meningococci, *Neisseria gonorrhoeae* and other bacteria. We identified the presence of a putative conserved PPIC-type peptidyl prolyl *cis/trans* isomerase (PPIase) domain (interval 133–259) belonging to the Rotamase_2 superfamily (Fig 1A and 1B). From BLAST analysis, NMB0345 shares 25.3% identity (= 73/288 amino acids) with the *C*. *jejuni* PEB3/CBF2 protein (273 amino acids, *Mr* 30.5kDa). NMB0345 can be putatively modelled in SWISS-model using as a template the crystallized structure of 3rfw.1. A protein (PEB4), which is the most closely related structure to *C*. *jejuni* PEB3/CBF2 that we have available in Protein Data Bank (Fig 1B). The model predicts a globular domain that contains the putative conserved PPIC-type PPIase domain (Val_133_-Lys_259_) extending from a ‘stalk’ of α-helices.

### Cloning, expression and purification of rCBF

**Fig 2:**
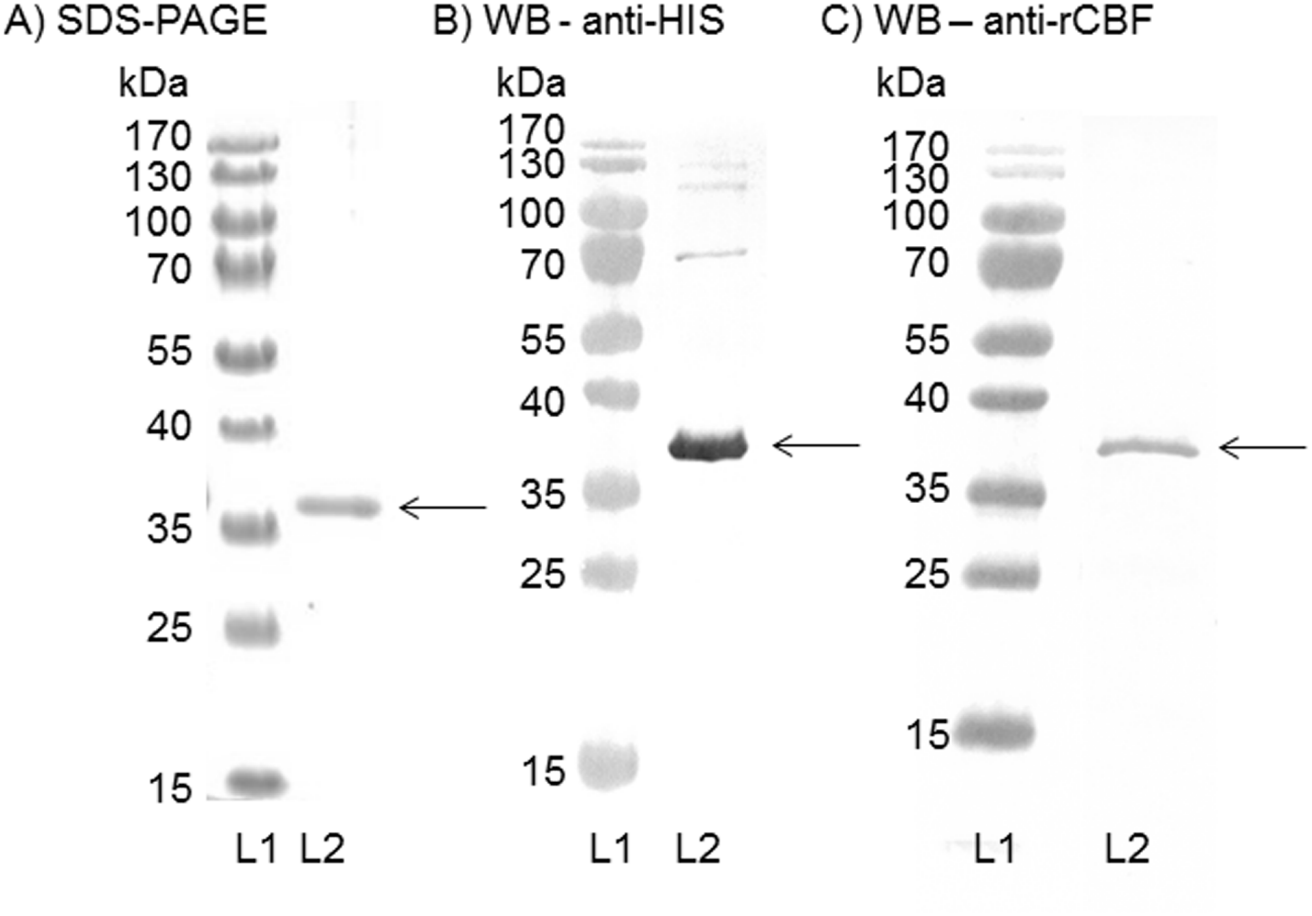
Purification of recombinant (r)CBF. The protein was purified to homogeneity using nickel-nitrilotriacetic acid metal affinity chromatography under denaturing conditions. **A) SDS-PAGE analysis of purified rCBF**. Lane 1, molecular mass markers; lane 2, rCBF (2 μg) showing a single band of ~36 kDa. **B) Western blot analysis of purified rCBF reacted with anti-HIS tag antibody**. Lane 1, molecular mass markers. Lane 2, protein reactivity with anti-HIS antibody (1/500) showing a single band of ~36 kDa. **C) Western blot analysis of purified rCBF reacted with rabbit polyclonal anti-rCBF serum**. Lane 1, molecular mass markers. Lane 2, protein reactivity with rabbit anti-rCBF serum (1/400) showing a single band of ~36 kDa.

Expression of rCBF protein from the pRSETA-*cbf* vector transformed into *E*.*coli* BL21(DE3) pLysS was induced with isopropyl-β-d-thiogalactopyranoside and *E*. *coli* bacteria were collected by centrifugation and lysed. The lysate was centrifuged to produce a supernatant and cell pellet and SDS-PAGE analysis demonstrated the presence of rCBF protein predominantly in the insoluble fraction, necessitating purification under denaturing conditions. SDS-PAGE analysis (Fig 2A) revealed a single band of *Mr* ~36 kDa, corresponding to full-length CBF protein with an N-terminal sequence of 39 additional amino acids including the 6×HIS tag. Recombinant protein reacted specifically in immunoblot with anti-HIS tag antibody and rabbit anti-rCBF sera (Fig 2B and 2C) as a single band of *Mr* ~36 kDa.

### rCBF protein induces humoral antibody responses in animals

**Fig 3:**
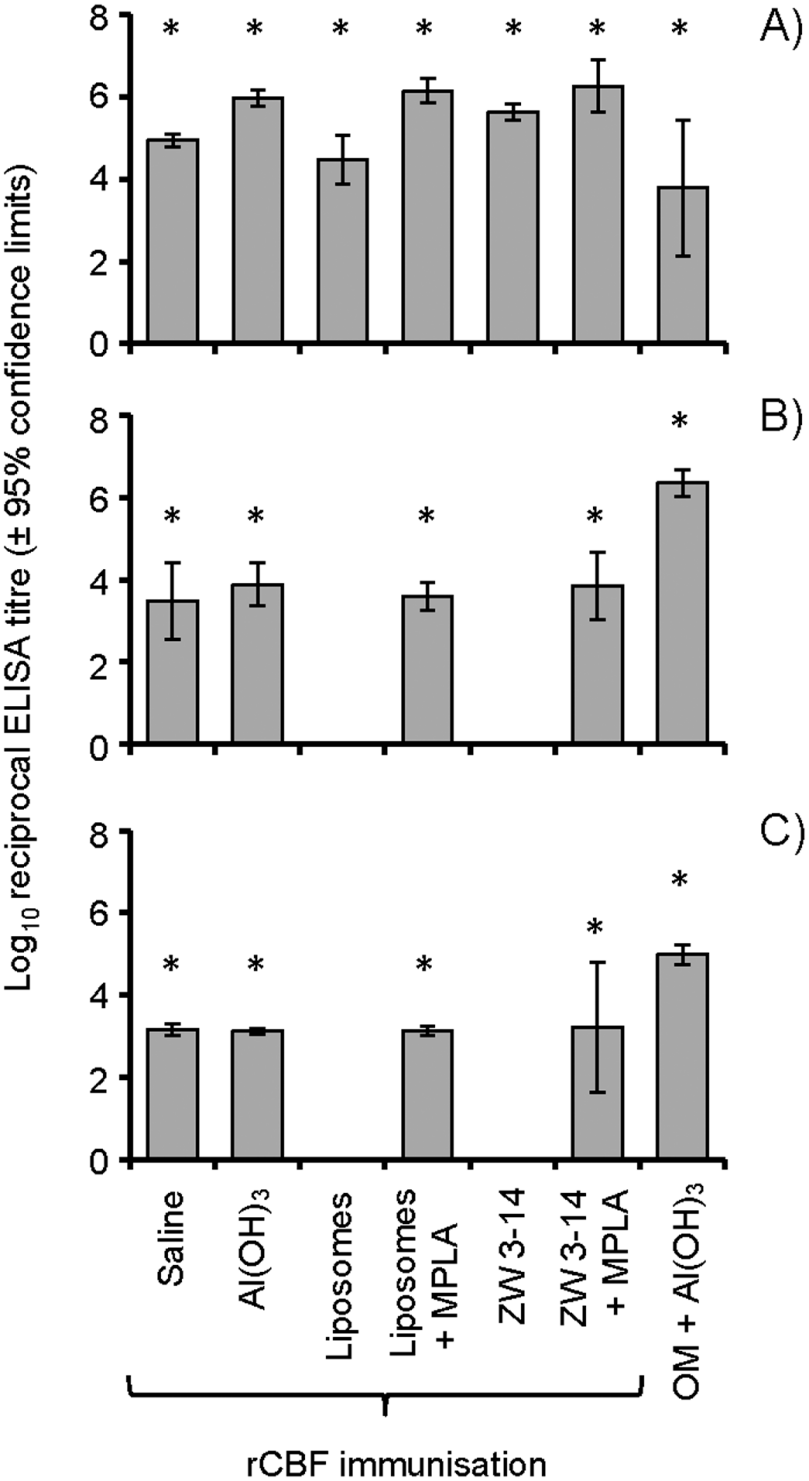
ELISA reactivity of antisera raised to different rCBF formulations against pure rCBF protein, outer membranes (OM) and whole meningococcal cells. Antisera from individual animals immunized with rCBF in various adjuvant and delivery formulations and from animals immunised with OM, were reacted in ELISA against **A) purified rCBF protein, B) MC58 OM and C) whole MC58 meningococcal cells**. The columns represent the geometric mean reciprocal ELISA titres (n = 4 or 5 animals per group) and the error bars represent the 95% confidence limits. No significant reactivity with rCBF, OM or whole cells in ELISA was observed with sera from sham-immunized animals or with normal mouse serum (absorbance values λ_450_nm <0.1 for serum dilutions of 1/10). The * denotes that the responses were statistically higher than the corresponding sham-immunized animals (p<0.05), using an independent t-Test to compare differences between the mean values.

ELISA of murine antisera raised against rCBF demonstrated that high (mean reciprocal ELISA) titres of antibodies were induced by immunisation with rCBF-saline (~88,000) and rCBF-liposomes (~30,000) (Fig 3A). Statistically higher (p<0.05) titres were induced by rCBF- Al(OH)_3_ (~934,000) or rCBF-Zw 3–14 micelles (~430,000) compared to the latter. Addition of MPLA into rCBF-liposomes and Zw 3–14 micelle preparations significantly increased serum antibody titres, with mean reciprocal ELISA titres ~1.5–2 × 10^6^ (p<0.05). Immunisation with MC58 OM adsorbed to Al(OH)_3_ induced lower levels of antibodies (mean titre ~6,000) that reacted with purified rCBF protein (Fig 3A).

The murine anti-rCBF antisera were also tested in ELISA against MC58 OM (Fig 3B) and whole meningococcal cells (Fig 3C). Weak reactivity with both preparations was observed with antisera raised to rCBF in saline, Al(OH)_3_ and in liposomes and Zw 3–14 micelles, both with MPLA. In general, mean reciprocal ELISA titres were significant (p<0.05) but low (ranging from ~1500–7000) and with variability in the responses of individual animals within each group. No reactivity was observed with antisera raised to rCBF in liposomes or Zw 3–14 micelles alone (Fig 3B and 3C). As expected, the reactivity of murine anti-OM sera against both MC58 OM and whole bacterial cells in ELISA was significantly higher than any of the murine antisera (p<0.05), with mean reciprocal ELISA titres of ~230,000 (Fig 3B) and ~100,000 (Fig 3C), respectively. Rabbit antisera raised to MC58 rCBF in Freund’s adjuvants was also tested in ELISA. Pre-immune rabbit serum did not react with purified rCBF (λ_450_nm absorbance values <0.1 for serum dilutions of 1/10) whereas post-immune rabbit serum demonstrated reciprocal ELISA titres ~130,000 against the protein. In addition, pre-immune rabbit serum showed no significant reactivity with both MC58 OM and whole bacterial cells in ELISA (titre <1000), whereas post-immune rabbit anti-rCBF serum reacted with OM and whole bacterial cells with reciprocal ELISA titres of 128,000 and 112,000 respectively. The hyper-reactivity of rabbit antiserum is likely a reflection of the potency of the Freund’s adjuvant and the dose and immunization schedule used for rCBF antigen in rabbits.

**Fig 4:**
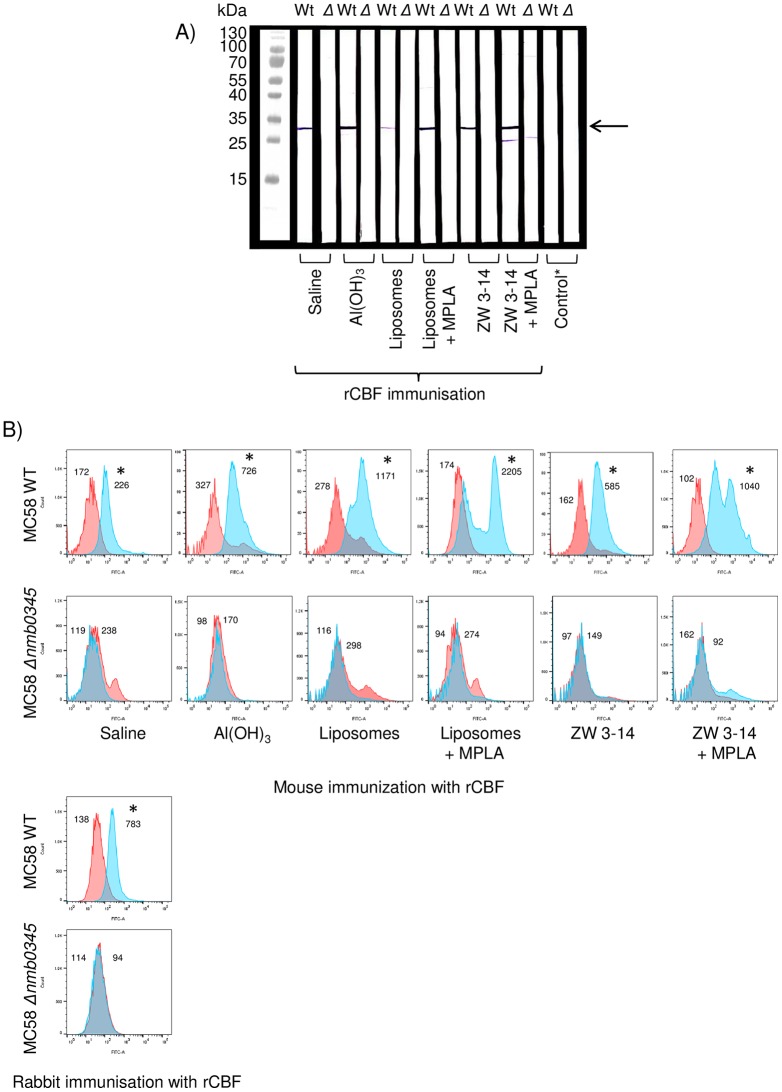
**A)** S**pecificity of murine anti-rCBF sera, examined by western immunoblot reactivity with CBF protein present in MC58 lysates**. Pooled antisera (1/100 dilution) were reacted against wild-type MC58 lysate (Wt) and MC58 *Δnmb0345* knock-out lysate (*Δ*) in western blot. CBF was recognised as a single protein band of *Mr* ~29.5 kDa (identified by the arrow) in wild-type MC58 (CBF^+^) but not in the knock-out strain (CBF^-^). All sham immunization sera and normal mouse serum were non-reactive against both Wt and knock-out strains, as demonstrated by representative strip blots (control*). **B) CBF is expressed on the surface of MC58 meningococci as demonstrated by FACS analysis**. On the top panel, the area within the red lines show no reactivity of wild-type MC58 (CBF^+^) bacteria with murine sham-immunised serum (1/10) and the area within the blue lines shows the significant FACS reactivity of murine antisera (1/10) raised against rCBF in the various formulations. The lower panel shows corresponding reactivity of the same murine sham-immunised sera and antisera against the MC58 *Δnmb0345* (CBF^-^) knock-out strain. Rabbit post-immune serum (1/100) also reacted with the wild-type MC58 (CBF^+^) (area within the blue line), compared to pre-immune serum (area within the red lines) and no reactivity was observed with the MC58 *Δnmb0345* (CBF^-^) knock-out strain. The numbers within each panel refer to the FITC-mean value. The * denotes the significant (p<0.05) and right-shifted increases in FITC-fluorescence recorded events, using a one sample t-Test to compare mean fluorescence values of test murine antisera against sham-immunised murine sera or rabbit post-immune antiserum against pre-immune serum. Data are representative of n = 2 experiments.

Western immuno-blotting demonstrated specificity of the murine immune responses (Fig 4A): antisera to rCBF-saline, rCBF-Al(OH)_3_, rCBF-liposomes ± MPLA and rCBF-Zw 3–14 micelles ± MPLA reacted with a single protein band of *Mr* ~29.5 kDa, which is consistent with homologous CBF protein in MC58 OM, whereas sera from sham-immunised mice were non-reactive. Specificity was confirmed by the lack of reactivity of murine antisera with the MC58 *Δnmb0345* knock-out strain that does not express CBF protein in the OM (Fig 4A). Murine antisera raised to MC58 OM adsorbed to Al(OH)_3_ was also tested in western blot against MC58 Wt and MC58 *Δnmb0345* knock-out strains as shown in S1 Fig: as expected, the position of the CBF band cannot be resolved from the complex multiple protein reactivity patterns for both preparations.

### Antibodies recognise CBF on MC58 meningococci

Antisera to rCBF in the various formulations reacted with MC58 (CBF^+^) bacteria, showing significant (p<0.05) and right-shifted increases in FITC-fluorescence recorded events, compared to sham-immunised murine sera (Fig 4B). Post-immune rabbit antiserum also showed significant (p<0.05) and right-shifted increase in FITC fluorescence-recorded events, compared to pre-immune serum. Specificity was confirmed by the lack of reactivity (P>0.05) of all murine antisera and rabbit post-immune serum with the MC58 *Δnmb0345* knock-out (CBF^-^) strain (Fig 4B).

In the western blot of antisera raised to rCBF-Zw 3–14 micelles + MPLA tested against the wild-type MC58, reactivity was also observed against a second minor band of *Mr* ~25kDa (Fig 4A), which was lower than the reactivity of this antiserum pool against the CBF protein (~29kDa). In addition, a second flow cytometry peak was observed when this antiserum pool was reacted against wild-type MC58, but not against the knock-out strain (Fig 4B). This cross-reactivity was not due to contamination of the rCBF used for immunization (Fig 2), since antisera raised to rCBF with any of the other preparations did not demonstrate a similar minor band (Fig 4A). This secondary minor band likely represents some murine antibody cross-reactivity with MC58, but it does not contribute to bactericidal activity, since no killing of the MC58*Δnmb0345* strain was observed with the bactericidal antisera raised to rCBF-Zw 3–14 micelles + MPLA (Table 1 below) using the baby rabbit complement serum bactericidal assay.

**Table 1:** Serum bactericidal activity of murine anti-rCBF sera.

			SBA titre induced by rCBF in ZW 3–14 + MPLA	
Strain	Serogroup	NMB0345 Allele	Baby rabbit complement	Human complement
MC58	B	1	256 (128, 256)	<4
MC168	B	1	1024	256
MC90	B	1	-	64
MC54	B	1	-	<8
M15 240010	W	1	-	8
M15 240190	Y	1	-	<8
L2470	B	18	256 (128, 256)	16
M15 240016	B	18	-	<8
MC162	C	3	<4	-
MENC11	C	3	<4	-
M15 240120	B	2	-	<8
M15 240106	B	2	-	<8

Bactericidal activity was determined with both baby rabbit serum and human Ig-depleted serum as exogenous complement sources. Titres for antisera raised to rCBF-saline, rCBF-Al(OH)_3_, rCBF-liposomes with and without MPLA, and rCBF-Zwittergent (Zw) 3–14 micelles alone as well as normal mouse serum and sera from sham immunized mice using either baby rabbit (BRC) or human complement (HC) sources, where tested, were not bactericidal (<4 against MC58). In addition, anti-rCBF in ZW 3–14 + MPLA sera did not kill the MC58 *Δnmb0345* knock-out strain (titre <4 using BRC). Data are the median values, with the range of values in parenthesis, from two-three independent measurements of bactericidal activity of all pooled serum samples. Single values denote that the bactericidal titres were identical from independent experiments.

- denotes not done for strains with BRC or HC, where no killing was observed with either assay.

### CBF is highly conserved and expressed in meningococci

The DNA sequences of the *cbf* gene (NEIS1825) from *N*. *meningitidis* isolates in the pubmlst.org/*Neisseria* database [31]—which contains data for an increasing collection of isolates representing the total known diversity of *Neisseria* species—were translated to amino acid sequences and aligned. In a combined total of 6644 *N*. *meningitidis* strains in the database with defined alleles, there are 141 Sequence Types (ST) represented. Using genome comparator, only 6/6644 (<0.1%) of meningococcal isolates lacked the *nmb0345* gene (id: 21459, 37778, 40426 (NG, non-groupable); 26977 (ND, not determined); 36468 (MenC); 40270 (MenW)), although it is possible that their absence may reflect errors in gene sequencing. There were 136 different allelic loci for which isolates were identified, and analysis of the translated proteins encoded by the nucleotide sequences of these different alleles identified 49 non-redundant NMB0345 amino acid sequences (S2 and S3 Figs, S1 Table). Expression of four different CBF proteins provided ~98% coverage of *N*. *meningitidis* isolates worldwide: the majority of meningococci expressed a representative protein encoded by Allele 1 (75% (4976/6644) of isolates) and this protein was found in MenA, MenB, MenC, MenE, MenH, MenW, MenX, MenY and MenZ isolates (S1 Table). *N*. *meningitidis* also expressed representative CBF proteins encoded by Alleles 18 (9%, 588/8844 isolates), 3 (9%, 599/6644 isolates) and 2 (5%, 303/6644 isolates) and these were found mainly in MenB and MenC isolates (S1 Table). In MenB isolates specifically, 23 CBF proteins with different amino acid sequences were found, with the remaining proteins expressed principally by MenY isolates with the occasional MenA, MenC, MenW and MenZ isolate (S1 Table). There were 2692 MenB isolates (41% of all meningococci) and 99% of these isolates were covered by CBF proteins representative of four Alleles: ~68% expressed representative protein encoded by Allele 1 (1833 isolates), 20% by Allele 18 (541 isolates), 8% by Allele 2 (213 isolates) and 3% by Allele 3 (72 isolates).

Examination of UK data collated during 2013–2015 for *N*. *meningitidis* strains in the database showed that there were 1537 isolates recorded (S2 Table). The majority of *N*. *meningitidis* isolates (90%) expressed representative CBF protein encoded by Allele 1 (79%, 1212/1537 isolates), which was found broadly expressed by MenB, MenC, MenW and MenY isolates, followed by Allele 18-encoded protein (11%, 164/1537 isolates), which was principally expressed by MenB strains. There were 794 MenB isolates recorded (52% of all meningococci), with the majority expressing a representative CBF protein encoded by Allele 1 (70%, 554/794 isolates) or Allele 18 (20%, 158/794 isolates,).

**Fig 5:**
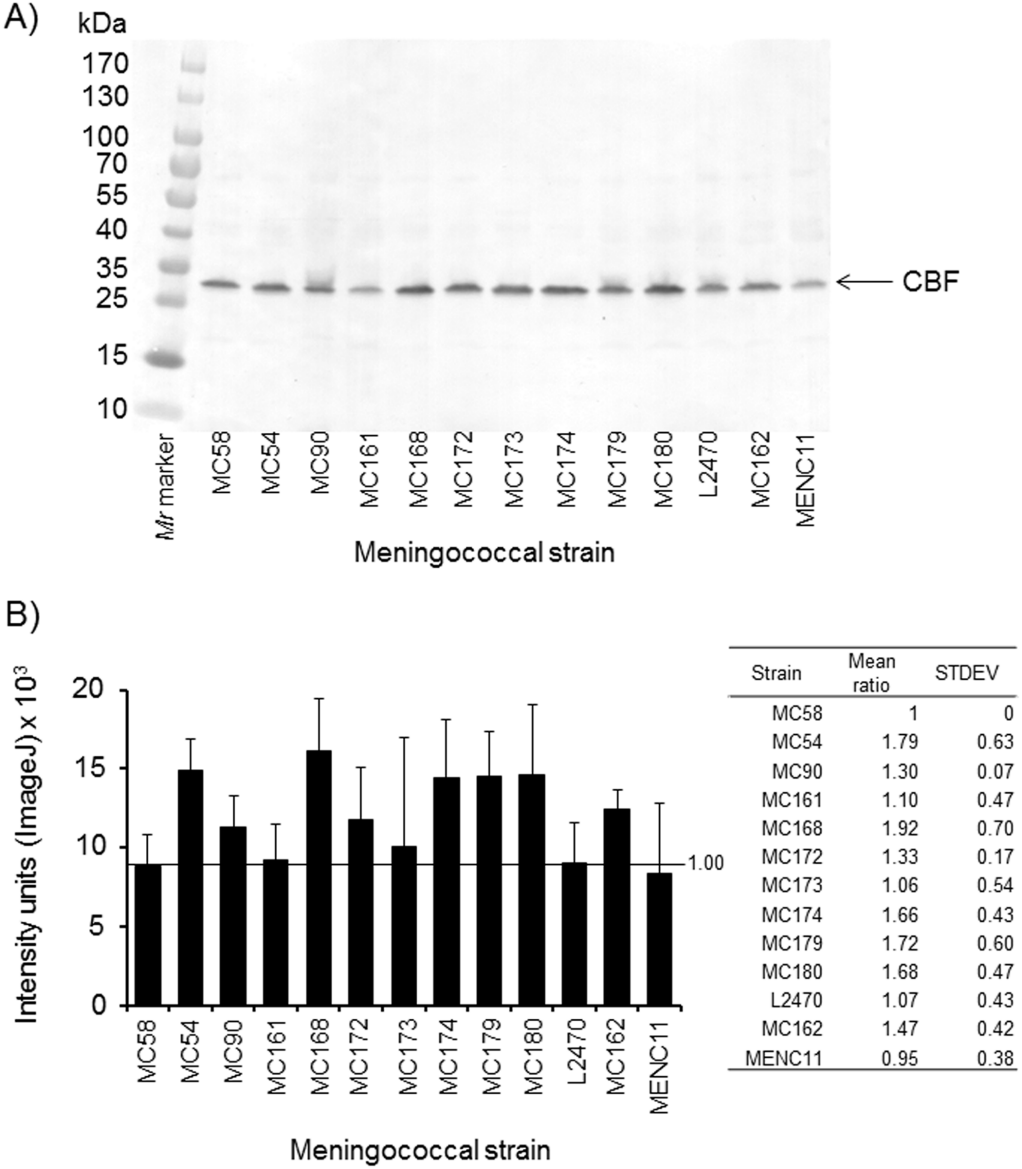
Production of CBF in different meningococcal strains examined by western blotting. **A)** Western blot reactivity of rabbit anti-rCBF sera (1/100 dilution) with CBF in lysates (10 μg of total protein) of meningococcal strains MC58, MC54, MC90, MC161, MC168, MC172, MC173, MC174, MC179 and MC180 (CBF encoded by Allele 1); L2470 (protein encoded by Allele 18) and MC162 and MENC11 (protein encoded by Allele 3). The blot is representative of n = 4 independent western blot experiments. **B)** For each of the blots, band intensities were quantified by ImageJ and a mean value for each meningococcal strain was calculated with standard deviation (for the bands from the n = 4 independent blots) shown by the error lines, as described previously [32]. For each blot, a ratio of band intensity relative to MC58 was also calculated for each strain and the mean ratio values with standard deviations (n = 4) are tabulated and compared with a student’s t-Test (p values >0.05 for all strains relative to MC58).

To investigate CBF protein production amongst different meningococcal strains, bacterial lysates from the 13 isolates from colonized individuals and from patients collected during an outbreak of meningococcal disease [18, 25] were reacted with rabbit anti-rCBF serum in western blots. Translation of the *nmb0345* gene sequences for these 13 isolates showed that they expressed protein sequences encoded by Allele 1(MC58, MC54, MC90, MC161, MC168, MC172, MC173, MC174, MC179, MC180), Allele 18 (L2470) or Allele 3 (MC162, MENC11). Examination of the amino acid sequences for these three CBF proteins showed high conservation, with only 2 amino acid differences observed; Val_138_ (Alleles 1, 3) to Ile_138_ (Allele 18) and Lys_176_ (Allele 1, 18) to Arg_176_ (Allele 3) (S2 Fig). Anti-rCBF sera reacted with a ~29.5kDa band present in all isolates (Fig 5A). The band intensities were also quantified from the blots using ImageJ software and appeared to be essentially similar (Fig 5B), with calculated mean ratios relative to MC58 between 1–2 (p>0.05).

### Bactericidal activity of anti-rCBF sera

Initially, murine antisera were tested for their ability to promote killing of meningococcal strain MC58 (encoding protein representative of Allele 1) in the presence of baby rabbit complement (BRC, 5% v/v) (Table 1). Only sera raised against rCBF-Zw 3–14 micelles + MPLA showed significant bactericidal activity against MC58 (reciprocal median titre 256 (64, 256), p<0.05) and similar cross-strain bactericidal activity (reciprocal median titres of 256 (128, 512)) against L2470, which expresses CBF encoded by Allele 18. To demonstrate specificity, rCBF-Zw 3–14 micelles + MPLA antisera did not kill the MC58 *Δnmb0345* knock-out strain (titres <4) in the presence of BRC. Sera from sham immunized animals and normal mouse serum did not induce bactericidal activity (<4).

Bactericidal activity of rCBF-Zw 3–14 micelles + MPLA antiserum pool was then examined using Ig-depleted human serum as a complement source (HC) in the more stringent hSBA assay (25% v/v) (Table 1). However, no killing was observed against homologous strain MC58 (titre <4). Therefore, we tested this antiserum pool in the hSBA against other strains that express protein encoded by Alelle 1: strains MC168 (MenB), MC90 (MenB) and M15 240010 (MenW) were killed by this antiserum pool (median titres of 256, 64 and 8 respectively), but not strains MC54 (MenB) or M15 240190 (MenY) (Table 1). For strains expressing heterologous protein encoded by Allele 18, this antiserum pool killed strain L2470 (MenB) in the hSBA with median titre 16, but not strain M15 240016 (MenB). No bactericidal activity (<4) was observed for strains MC162 and MENC11 (both MenC strains), expressing heterologous CBF encoded by Allele 3 with BRC, so these were not tested with HC (Table 1). We also tested this antiserum pool against two MenB strains expressing protein encoded by Allele 2 (M15 240120 and M15 240106) but no bactericidal activity was observed with HC (Table 1).

## Discussion

To our knowledge, this study is the first to demonstrate that a recombinant putative Cell Binding Factor protein (CBF, NMB0345/NEIS1825) can induce murine antibodies with bactericidal activity against meningococci. The protein has been reported in several meningococcal OM/OMV proteomes [26, 33–35] and despite PSORTb (3.0) failing to assign CBF to a bacterial compartment, we provide evidence that the protein appears to be surface-accessible on MC58 bacteria. CBF belongs to the meningococcal immuno-OM proteome as reported in our initial study [18], and in our current study, we demonstrated the reactivity of murine and rabbit anti-rCBF sera with OM and whole meningococcal cells. The demonstrable bactericidal activity of anti-rCBF sera for wild-type meningococci but not for a CBF knock-out strain suggests that CBF is accessible on the surface of the bacterium to binding antibodies. This is confirmed by the FACS reactivity of anti-rCBF sera with wild-type MC58 but not mutant meningococci: indeed, FACS has been often used as an indicator of surface-accessibility of bactericidal antibodies and as a selection tool for candidate *Neisseria* vaccine antigens identified by *in silico* reverse vaccinology [5, 36].

We were also able to generate a putative model of the CBF protein based on the most closely related *C*. *jejuni* PEB3/CBF2, using the PEB4 homologue as a template. The *C*. *jejuni* homologue is a putative periplasmic protein, but examination of the amino acid sequences showed that the meningococcal CBF protein shares only 73/288 amino acids with PEB3/CBF2 protein, and that this similarity is evenly spread across the protein sequences. Moreover, the meningococcal CBF protein has a globular domain, which contains the putative conserved PPIC-type PPIase domain, which is similar in structure to the globular domains found in the Macrophage Infectivity Potentiator (MIP)-like PPIase proteins that are expressed in the OM of the *Neisseriae* and other pathogens [37]. It is possible that meningococcal CBF does not function as a periplasmic chaperone like the predicted function for *C*. *jejuni* PEB3/CBF2, but rather is an OM-located, surface-exposed molecule with PPIase function like the MIP proteins. Taken together, the observations that the meningococcal CBF shows only ~25% identity with the periplasmic PEB3/CBF2 protein, has structural similarity to *Neisseria* MIP protein(s), is a putative lipoprotein with a cleavable leader peptide and is capable of binding bactericidal antibodies, suggests that the CBF protein is probably OM-located and surface-accessible.

Interrogation of NMB0345/NEIS1825 amino acid sequences from isolates reported in the pubmlst.org/*Neisseria* database and our own strain collection demonstrated conservation of this protein across meningococci. We demonstrated using western blot reactivity with rabbit anti-rCBF serum that CBF was also produced by all strains examined thus far. rCBF could induce functional bactericidal antibodies, but only when delivered in detergent micelles containing exogenous MPLA adjuvant and surprisingly, not with several other adjuvants that are compatible for human immunisation. The lack of bactericidal activity induced by rCBF with these adjuvants did not correlate with the observations of production of high levels of specific antibodies that reacted with surface-exposed CBF on meningococci and may reflect differences in adjuvant-directed antigen processing and antibody avidity.

In the current study, we used initially the BRC SBA assay to examine bactericidal activity and then tested the antisera in the more stringent human (h)SBA assay. The limitation of our study was that finite serum volumes with repeated experiments to demonstrate reproducibility, allowed only for a restricted number of strains expressing CBF proteins encoded by different alleles to be tested in the SBA assays. Using the BRC SBA assay, we demonstrated initially that anti-rCBF sera raised in Zw 3–14 micelles with MPLA killed the homologous strain MC58 (CBF encoded by Allele 1) and the heterologous strain L2470 (expressing CBF encoded by Allele 18 with >99% identity to the MC58 variant). However, no killing was observed with the heterologous strains expressing CBF encoded by Allele 3. Nevertheless, and assuming that all strains were killed in the same manner, by simple extrapolation, a single protein could potentially cover worldwide at least ~84% of the total meningococcal strains in the pubmlst.org/*Neisseria* database. Notably, proteins encoded by Allele 1 can be found in all the major serogroups (A, B, C, Y and W) causing disease. Furthermore, at least 88% of all MenB strains worldwide in the database would be covered by immunization with a single protein capable of inducing cross-strain bactericidal activity (S1 Table). An analysis of disease occurring specifically in the UK, demonstrated that at least 90% of MenB isolates (n = 712/794) causing disease in recent years (2013–2015) would be covered by using a cross-protective rCBF protein encoded by Allele 1 and coverage could extend potentially to disease caused by the majority of MenC, MenW and MenY isolates recorded (n = 643 isolates, S2 Table).

From this preliminary screen with a limited number of strains, we extended our study to examine whether the anti-rCBF sera were bactericidal in the more stringent hSBA assay. However, this assay highlighted variability between strains in their ability to be killed by anti-rCBF sera, which we have observed in a previous study with another candidate vaccine protein, rNMB1612 [32]. We observed no killing of MC58 or MC54 MenB strains expressing protein encoded by Allele 1 in the hSBA assay: by contrast, two other MenB strains, MC168 and MC90, expressing the same Allele 1 protein, were killed by rCBF antibodies using HC. The lack of killing of MC58 and MC54 was not due to differences in CBF production as judged by western blot reactivity and explanations for variability between strains in susceptibility to killing are afforded by our rNMB1612 study. Furthermore, variability could also be linked to surface-exposure of CBF [32]: to test this hypothesis, we used the rabbit anti-rCBF serum (with high titre reactivity with OM and meningococcal cells and cross-reactivity with CBF proteins encoded by different alleles) to examine by FACS the surface-exposure of CBF in the different meningococcal strains. We observed that antibodies were capable of binding to the surface of MC58 meningococci as well as to strains MC168, MC90 and L2470 (S4 Fig), but not to strains MC54, M15 240010, M15 240190, M15 240016, MC162, MENC11, M15 240120 and M15 240106. Interestingly, bactericidal activity of murine antisera, whether with the BRC SBA or the hSBA was observed against the strains showing positive FACS reactivity for CBF expression, but was absent against the strains showing no significant FACS reactivity. Thus, it is possible that variable CBF transport and surface-exposure and structural differences in OM between strains could influence the binding of rCBF-induced antibodies to CBF bactericidal epitopes. Furthermore, structural differences that may influence the ability of antisera to kill meningococci with HC in particular, could include variable/different expression of capsule or fHbp protein, which prevents complement-mediated death, and perhaps the presence of truncated, non-sialylated lipooligosaccharide.

It is interesting to note that the anti-rCBF sera weakly killed a MenW strain (titre of 8) but not a MenY strain, each encoding the homologous CBF protein (Allele 1). With respect to heterologous SBA, anti-rCBF sera killed only one of two strains expressing CBF encoded by Allele 18 in the hSBA assay and did not kill either of two strains that expressed CBF encoded by Allele 2. Thus, extrapolation of these data to determine coverage rates worldwide and in the UK specifically can only be hypothetical: it is unclear whether lack of killing of different strains *in vitro* would be observed in humans, reflecting differences possibly in virulence, and emphasizes the need for testing bactericidal activity with the hSBA against much larger numbers of isolates expressing proteins encoded by the different Alleles. Thus, estimates of potential coverage of MenB using a rCBF protein encoded by Alelle 1 that cross-protects against strains expressing protein encoded by Allele 18, would be significantly down-graded if only 50% of strains circulating in the population behaved in the hSBA assay similarly as strain MC168, MC90 and L2470. A similar down-grading would be observed for MenY and MenW isolates expressing CBF encoded by Allele 1.

## Conclusion

CBF protein is produced with limited variation in meningococci and rCBF protein can induce cross-strain bactericidal antibodies. However, there appear to be differences in surface-exposure of the antigen between meningococcal strains. Further studies would be required to identify suitable, human-compatible adjuvants for use with this protein. Most importantly, candidature for inclusion of rCBF in next-generation MenB or ‘universal’ vaccines will depend on demonstration of an ability to elicit bactericidal antibodies against a much larger set of strains representative of meningococcal strain diversity associated with meningococcal infections.

## Supporting Information

S1 Fig Western blot reactivity of murine antisera raised against MC58 outer membranes adsorbed to Al(OH)_3_. Strip blots containing 5μg of protein were each reacted with pooled murine antiserum (1/200 dilution). Wt denotes wild-type MC58 (CBF^+^) and *Δ* denotes the MC58 *Δnmb0345* (CBF^-^ knock-out) preparation tested.
(PPTX)

S2 Fig Alignment of non-redundant NMB0345 (NEIS1825) amino acid sequences for *Neisseria meningitidis* isolates in the http://pubmlst.org/perl/bigsdb/bigsdb.pl?db=pubmlst_neisseria_isolates database and the additional 13 strains in our collection. Database was accessed 01-03-2016. Amino acid sequence alignments were generated using Clustal (http://www.ebi.ac.uk/Tools/msa/clustalo/) and a dendrogram was then assembled using the non-redundant sequences with Jalview 2.8 (www.jalview.org). *Denotes identical amino acids;—denote position of amino acid change. A denotes Allele.
(DOCX)

S3 Fig A dendrogram showing the relationships between the 49 non-redundant NMB0345 (NEIS1825) alleles in *Neisseria meningitidis* isolates in the pubmlst.org/*Neisseria* database. The numbers denote the average distance using % identity tree calculated by Jalview. A denotes Allele.
(PPTX)

S4 Fig FACS reactivity of rabbit pre-immune and anti-rCBF serum with meningococcal strains. Pre-immune (red line) and post-immune (blue line) serum raised to rCBF in Freund’s adjuvants was tested (1/100 dilution) in FACS against *Neisseria meningitidis* strains used for serum bactericidal assays. Significant (p<0.05) right-sided shifts in FITC-recorded events was shown for strains MC168, MC90 and L2470, whereas no significant right-sided shifts were observed for strains MC54, M15 240010, M15 240190, M15 240016, MC162, MENC11, M15 240120 and M15 240106 (plots not shown). Positive reactivity for MC58 is shown in Fig 4B.
(PPTX)

S1 Table Analysis of NMB0345 (NEIS1825) alleles and number of isolates per serogroup: data are collated from http://pubmlst.org/perl/bigsdb/bigsdb.pl?db=pubmlst_neisseria_isolates and also include the 13 strains from our collection. Numbers in parentheses indicate that the Alleles produce proteins with identical amino acid sequences. Database was accessed 01-03-2106 and there are 136 allelic loci with isolates generating 49 non-redundant protein amino acid sequences. NG, no serogroup identified; ND, not determined. Table sorted numerically according to Alleles containing similar allelic proteins and then single Alleles.
(DOCX)

S2 Table Analysis of NMB0345 (NEIS1825) alleles and number of isolates per serogroup for UK data 2013–2015: data are collated from http://pubmlst.org/perl/bigsdb/bigsdb.pl?db=pubmlst_neisseria_isolates. Numbers in parentheses indicate that the Alleles produce proteins with identical amino acid sequences. Database was accessed 01-03-2016 for the UK from 2013–2015, the most recent data. NG, no serogroup identified; ND, not determined.
(DOCX)
