# Supplementary figures and images for: Vaccine Potential and Diversity of the Putative Cell Binding Factor (CBF, NMB0345/NEIS1825) Protein of Neisseria meningitidis
Source: PLoS One. 2016 Aug 9;11(8):e0160403. doi: 10.1371/journal.pone.0160403 (PMC4978444; doi:10.1371/journal.pone.0160403)

## Slide 1
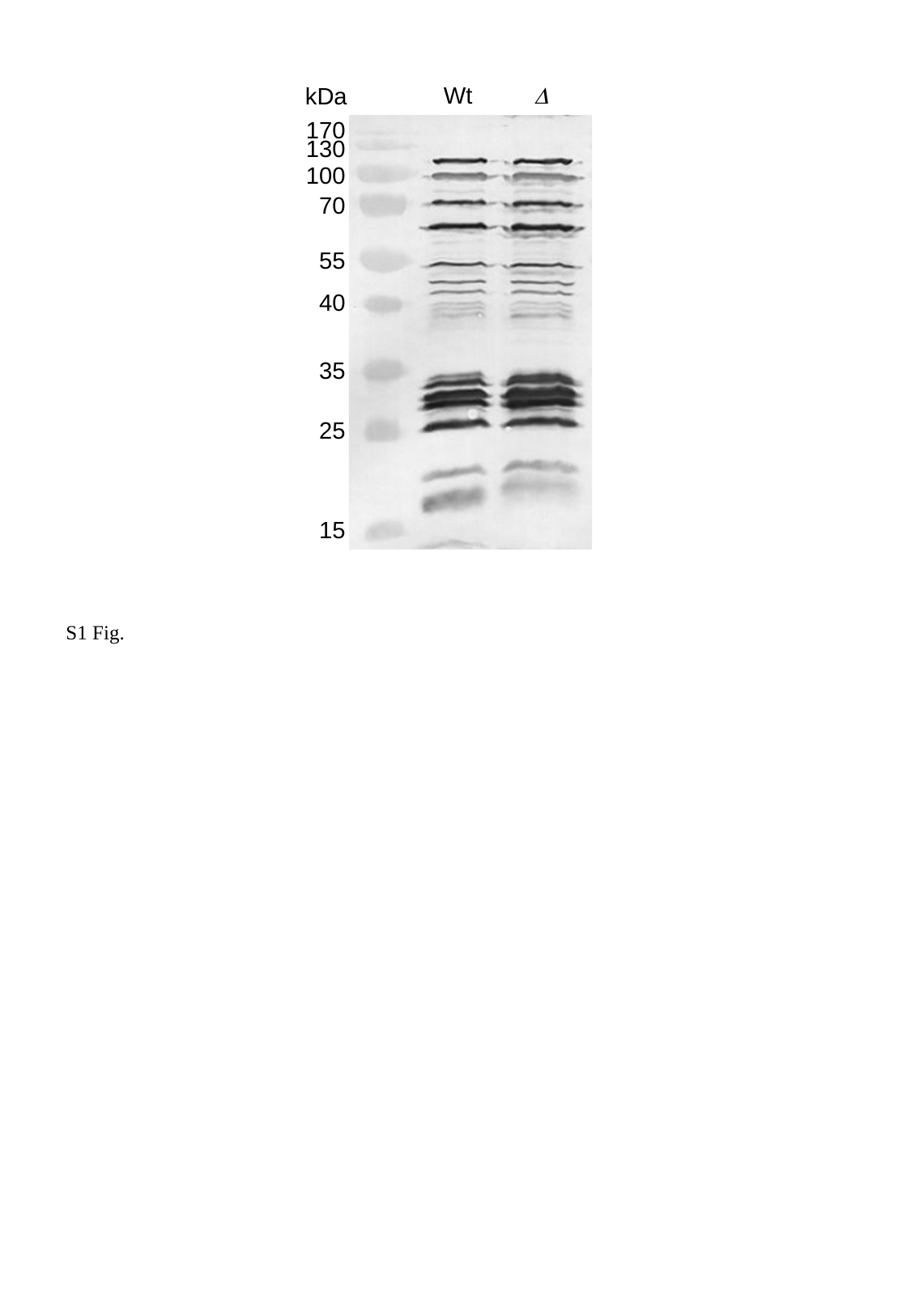

Δ
Wt
kDa
170
130
100
70
55
40
35
25
15
S1 Fig.

Supplement: S1 Fig — Strip blots containing 5μg of protein were each reacted with pooled murine antiserum (1/200 dilution). Wt denotes wild-type MC58 (CBF+) and Δ denotes the MC58 Δnmb0345 (CBF- knock-out) preparation tested. (PPTX) [file pone.0160403.s001.pptx]

## Slide 1
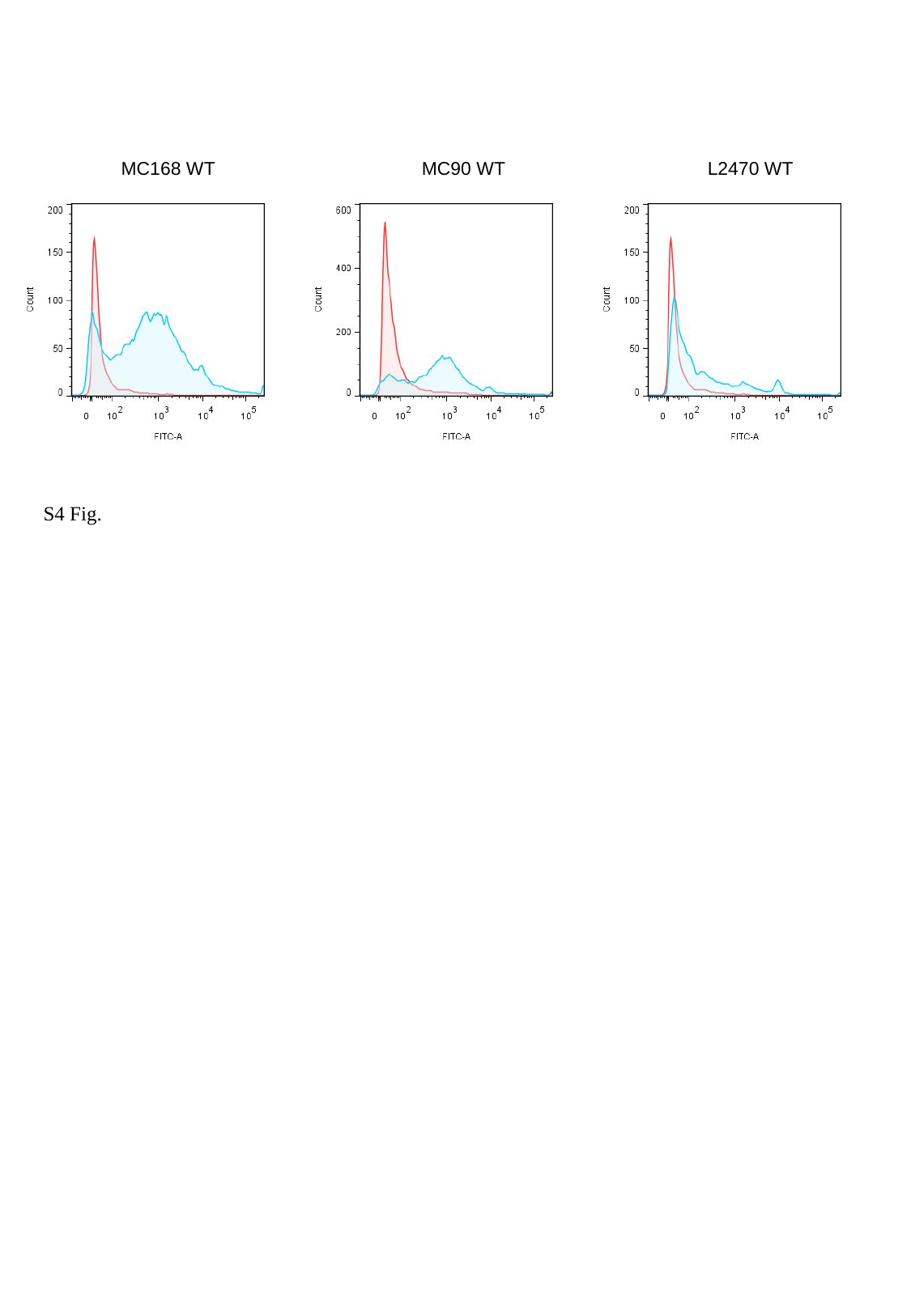

MC168 WT
L2470 WT
MC90 WT
S4 Fig.

Supplement: S4 Fig — Pre-immune (red line) and post-immune (blue line) serum raised to rCBF in Freund’s adjuvants was tested (1/100 dilution) in FACS against Neisseria meningitidis strains used for serum bactericidal assays. Significant (p<0.05) right-sided shifts in FITC-recorded events was shown for strains MC168, MC90 and L2470, whereas no significant right-sided shifts were observed for strains MC54, M15 240010, M15 240190, M15 240016, MC162, MENC11, M15 240120 and M15 240106 (plots not shown). Positive reactivity for MC58 is shown in Fig 4B. (PPTX) [file pone.0160403.s004.pptx]
