# Supplementary material for: Vaccine Potential and Diversity of the Putative Cell Binding Factor (CBF, NMB0345/NEIS1825) Protein of Neisseria meningitidis
Source: PLoS One. 2016 Aug 9;11(8):e0160403. doi: 10.1371/journal.pone.0160403 (PMC4978444; doi:10.1371/journal.pone.0160403)
Supplement: S2 Fig — Database was accessed 01-03-2016. Amino acid sequence alignments were generated using Clustal (http://www.ebi.ac.uk/Tools/msa/clustalo/) and a dendrogram was then assembled using the non-redundant sequences with Jalview 2.8 (www.jalview.org). *Denotes identical amino acids;—denote position of amino acid change. A denotes Allele. (DOCX) [file pone.0160403.s002.docx]

A206 MKAKILTSVALLACSGSLFAQTLATVNGQKIDSSVIDAQVAAFRAENSSAEDSPQLRRAL 60

A36 MKAKILTSAALLACSGSLFAQTLATVNGQKIDSSVIDAQVAAFRAENSRAEDSPQLRRAL 60

A39 MKAKILTSVALLACSGSLFAQTLATVNGQKIDSSVIDAQVAAFRAENSSAEDSPQLRRAL 60

A40 MKAKILTSVALLACSGSLFAQTLATVNGQKIDSSVIDAQVAAFRAENSSAEDSPQLRRAL 60

A34 MKAKILTSVALLACSGSLFAQTLATVNGQKIDSSVIDAQVAAFRAENSSAEDSPQLRRAL 60

A204 MKAKILTSVALLACSGSLFAQTLATVNGQKIDSSVIDAQVAAFRAENSRAEDSPRLRQAL 60

A181 MKAKILTSVALLACSGSLFAQTLATVNGQKIDSAVIEAQVAAFRAENSRAEDTPQLRQSL 60

A216 MKAKILTSVALLACSGSLFAQTLATVNGQKIDSSVIDAQVAAFRAENSRAEDTPQLRQSL 60

A211 MKAKILTSVALLACSGSLFAQTLATVNGQKIDSSVIDAQVAAFRAENSRAEDTPQLRQSL 60

A197 MKAKILTSVALLACSGSLFAQTLATVNGQKIDSSVIDAQVAAFRAENSRAEDTPQLRQSL 60

A144 MKAKILTSVALLACSGSLFAQTLATVNGQKIDSSVIDAQVAAFRAENSRAEDTPQLRQSL 60

A140 MKAKILTSVALLACSGSLFAQTLATVNGQKIDSSVIDVQVAAFRAENSRAEDTPQLRQSL 60

A139 MKAKILTSVALLACSGSLFAQTLATVNGQKIDSSVIDAQVSAFRAENSRAEDTPQLRQSL 60

A191 MKAKILTSVALLACSGSLFAQTLATVNGQKIDSSVIDAQVAAFRAENSRAEDTPQLRQSL 60

A189 MKAKILTSVALLACSGSLFAQTLATVNGQKIDSSVIDAQVAAFRAENSRAEDTPQLRQSL 60

A130 MKAKILTSVALLACSGSLFAQTLATVNGQKIDSSVIDAQVAAFRAENSRAEDTPQLRQSL 60

A177 MKAKILTSVALLACSGSLFAQTLATVNGQKIDSSVIDAQVAAFRAENSRAEDTPQLRQSL 60

A106 MKAKILTSVALLACSGSLFAQTLATVNGQKIDSSVIDAQVAAFRAGNSRAEDTPQLRQSL 60

A168 MEAKILTSVALLACSGSLFAQTLATVNGQKIDSSVIDAQVAAFRAENSRAEDTPQLRQSL 60

A162 MKAKILTSVALLACSGSLFAQTLATVNGQKIDSSVIDAQVAAFRAENSRAEDTPQLRQSL 60

A161 MKAKILTSVALLACSGSLFAQTLATVNGQKIDSSVIDAQVAAFRAENSRAEDTPQLRQSL 60

A160 MKAKILTSVALLACSGSLFAQTLATVNGQKIDSSVIDAQVAAFRAENSRAEDTPQLRQSL 60

A123 MKAKILTSVALLACSGSLFAQTLATVNGQKIDSSVIDAQVAAFRAENSRAEDTPQLRQSL 60

A107 MKAKILTSVALLACSGSLFAQTLATVNGQKIDSSVIDAQVAAFRAENSRAEDTPQLRQSL 60

A78 MKAKILTSVALLACSGSLFAQTLATVNGQKIDSSVIDAQVAAFRAENSRTEDTPQLRQSL 60

A117 MKAKILTSVALLACSGSLFAQTLATVNGQKIDSSVIDAQVAAFRAENSRAEDTPQLRQSL 60

A116 MKAKILTSVALLACSGSLFAQTLATVNGQKIDSSVIDAQVAAFRAENSRAEDTPQLRQSL 60

A114 MKAKILTSVALLACSGSLFAQTLATVNGQKIDSSVIDAQVAAFRAENSRAEDTSQLRQSL 60

A108 MKAKILTSVALLACSGSLFAQTLATVNGQKIDSSVIDAQVAAFRAKNSRAEDTPQLRQSL 60

A97 MKAKILTSVALLACSGSLFAQTLATVNGQKIDSSVIDAQVAAFRAENSRAEDTPQLRQSL 60

A61 MKAKILTSVALLACSGSLFAQTLATVNGQKIDSSVIDAQVAAFRAENSRAEDTPQLRQSL 60

A58 MKAKILTSVALLACSGSLFAQTLATVNGQKIDSSVIDAQVAAFRAENSRAEDTPQLRQSL 60

A57 MKAKILTSVALLACFGSLFAQTLATVNGQKIDSSVIDAQVAAFRAENSRAEDTPQLRQSL 60

A94 MKAKILTSVALLACSGSLFAQTLATVNGQKIDSSVIDAQVAAFRAENSRAEDTPQLRQSL 60

A92 MKAKILTSVALLACSGSLFAQTLATVNGQKIDSSVIDAQVAAFRAENSRAEDTPQLRQSL 60

A73 MKAKILTSVALLACSGSLFAQTLATVNGQKIDSSVIDAQVAAFRAENSRAEDTPQLRQSL 60

A81 MKAKILTSVALLACSGSLFAQTLATVNGQKIDSSVIDAQVAAFRAENSRAEDTPQLRQSL 60

A79 MKAKILTSVALLACSGSLFAQTLATVNGQKIDSSVIDAQVAAFRAENSRAEDTPQLRQSL 60

A155 MKAKILTSVALLACSGSLFAQTLATVNGQKIDSSVIDAQVAAFRAENSRAEDTPQLRQSL 60

A153 MKAKILTSVALLACSGSLFAQTLATVNGQKIDSSVIDAQVAAFRAENSRAEDTPQLRQSL 60

A52 MKAKILTSVALLACSGSLFAQTLATVNGQKIDSSVIDAQVAAFRAENSRAEDTPQLRQSL 60

A27 MKAKILTFVALLACSGSLFAQTLATVNGQKIDSSVIDAQVAAFRAENSRAEDTPQLRQSL 60

A3 MKAKILTSVALLACSGSLFAQTLATVNGQKIDSSVIDAQVAAFRAENSRAEDTPQLRQSL 60

A18 MKAKILTSVALLACSGSLFAQTLATVNGQKIDSSVIDAQVAAFRAENSRAEDTPQLRQSL 60

A2 MKAKILTSVALLACSGSLFAQTLATVNGQKIDSSVIDAQVAAFRAENSRAEDTPQLRQSL 60

A12 MKAKILTSVALLACSGSLFAQTLATVNGQKIDSSVIDAQVAAFRAENSRAEDTPQLRQSL 60

A1 MKAKILTSVALLACSGSLFAQTLATVNGQKIDSSVIDAQVAAFRAENSRAEDTPQLRQSL 60

A208 MKAKILTSVALLACSGSLFAQTLATVNGQKIDSSVIDAQVAAFRAENSRAEDTPQLRQSL 60

A215 MKAKILTSVALLACSGSLFAQTLATVNGQKIDSSVIDAQVAAFRAENSRAEDTPQLRQSL 60

*:***** .***** ******************:**:.**:**** ** :**: :**::*

A206 LNQEITNTVVAQEVKRLKLDRSAEFKDMLAKLRAEAEKSGDDKKPSFKTVWQAVEYGLNG 120

A36 LNQEITNTVVAQEVKRLKLDRSAEFKDMLAKLRAEAEKSGDDKKPSFKTVWQAVEYGLNG 120

A39 LNQEITNTVVAQEVKRLKLDRSAEFKDMLAKLRAEAEKSGDDKKPSFKTVWQAVEYGLNG 120

A40 LNQEITHTVVAQEVKRLKLDRSAEFKDMLAKLRAEAEKSGDDKKPSFKTVWQAVEYGLNG 120

A34 LNQEITHTVVAQEVKRLKLDRSAEFKDMLAKLRAEAEKSGDDKKPSFKTVWQAVEYGLNG 120

A204 LENEVVNTVVAQEVKRLKLDQSAEFKDTLAKLRAEAKKSGDDKKPSFKTVWSAVEYELNG 120

A181 LENEVVNTVVAQEVKRLKLDRSAEFKNALAKLRAEAKKSGDDKKPSFKTVWQAVKYGLNG 120

A216 LENEVVNTVVAQEVKRLKLDRSAEFKNALAKLRTEAKKSGDDKKPSFKTVWQAVKYGLNG 120

A211 LENEVVNTVVAQEVKHLKLDRSAEFKNALAKLRAEAKKSGDDKKPSFKTVWQAVKYGLNG 120

A197 LENEVVNTVVAQEVKRLKLDRSAEFKNALAKLRAEAKKSGDDKKPSFKTVWQTVKYGLNG 120

A144 LENEVVNTVVAQEVKRLKLDRSAEFKNALAKLRAEAKKSGDDKKPSFKTVWQAVKYGLNG 120

A140 LENEVVNTVVAQEVKRLKLDRSAEFKNALAKLRAEAKKSGDDKKPSFKTVWQAVKYGLNG 120

A139 LENEVVNTVVAQEVKRLKLDRSAEFKNALAKLRAEAKKSGDDKKPSFKTVWQAVKYGLNG 120

A191 LENEVVNTVVAQEVKRLKLDRSAEFKNALAKLRAEAKKSGDDKKPSFKTVWQAVKYGLNG 120

A189 LENEVVNTVVAQEVKRLKLDRSAEFKNALAKLRAEAKKSGDDKKPSFKTVWQAVKYGLNG 120

A130 LENEVVNTVVAQEVKRLKLDRSAEFKNALAKLRAEAKKSGDDKKPSFKTVWQAVKYGLNG 120

A177 LENEVVNTVVAQEVKRLKLDRSAEFKNALAKLRAEAKKSGDDKKPSFKTVWQAVKYGLNG 120

A106 LENEVVNTVVAQEVKRLKLDRSAEFKNALAKLRAEAKKSGDDKKPSFKTVWQAVKYGLNG 120

A168 LENEVVNTVVAQEVKRLKLDRSAEFKNALAKLRAEAKKSGDDKKPSFKTVWQAVKYGLNG 120

A162 LENEVVNTVVAQEVKRLKLDRSAEFKNALAKLRAEAKKSGDDKKPSFKTVWQAVKYGLNG 120

A161 LENEVVNTVVAQEVKRLKLDRSAEFKNALAKLRAEAKKSGDDKKPSFKTVWQAVKYGLNG 120

A160 LENEVVNTVVAQEVKRLKLDRSAEFKNALAKLRAEAKKSGDDKKPSFKTVWQAVKYGLNG 120

A123 LENEVVNTVVAQEVKRLKLDRSAEFKNALAKLRAEAKKSGDDKKPSFKTVWQAVKYGLNG 120

A107 LENEVVNTVVAQEVKRLKLDRSAEFKNALAKLRAEAKKSGDDKKPSFKTVWQAVKYGLNG 120

A78 LENEVVNTVVAQEVKRLKLDRSAEFKNALAKLRAEAKKSGDDKKPSFKTVWQAVKYGLNG 120

A117 LENEVVNTVVAQEVKLLKLDRSAEFKNALAKLRAEAKKSGDDKKPSFKTVWQAVKYGLNG 120

A116 LENEVVNTVVAQEVKRLKLDRSAEFKNALAKLRAEAKKSGDDKKPSFKTVWQAVKYGLNG 120

A114 LENEVVNTVVAQEVKRLKLDRSAEFKNALAKLRAEAKKSGDDKKPSFKTVWQAVKYGLNG 120

A108 LENEVVNTVVAQEVKRLKLDRSAEFKNALAKLRAEAKKSGDDKKPSFKTVWQAVKYGLNG 120

A97 LENEVVNTVVAQEVKRLKLDRSAEFKNALAKLRAEAKKSGDDKKPSFKTVWQAVKYGLNG 120

A61 LENEVVNTVVAQEVKRLKLDRSAEFKNALAKLRAEAKKSGDDKKPSFKTVWQAVKYGLNG 120

A58 LENEVVNTVVAQEVKRLKLDRSAEFKNALAKLRAEAKKSGDDKKPSFKTVWQAVKYGLNG 120

A57 LENEVVNTVVAQEVKRLKLDRSAEFKNALAKLRAEAKKSGDDKKPSFKTVWQAVKYGLNG 120

A94 LENEVVNTVVAQEVKRLKLDRSAEFKNALAKLRAEAKKSGDDKKPSFKTVWQAVKYGLNG 120

A92 LENEVVNTVVAQEVKRLKLDRSAEFKNALAKLRAEAKKSGDDKKPSFKTVWQAVKYGLNG 120

A73 LENEVVNTVVAQEVKRLKLDRSAEFKNALVKLRAEAKKSGDDKKPSFKTVWQAVKYGLNG 120

A81 LENEVVNTVVAQEVKRLKLDRSAEFKNALAKLRAEAKKSGDDKKPSFKTVWQAVKYGLNG 120

A79 LENEVVNTVVAQEVKRLKLDRSAEFKNALAKLRAEAKKSGDDKKPSFKTVWQAVKYGLNG 120

A155 LENEVVNTVVAQEVKRLKLDRSAEFKNALAKLRAEAKKSGDDKKPSFKTVWQAVKYGLNG 120

A153 LENEVVNTVVAQEVKRLKLDRSAEFKNALAKLRAEAKKSGDDKKPSFKTVWQAVKYGLNG 120

A52 LENEVVNTVVAQEVKRLKLDRSAEFKNALAKLRAEAKKSGDDKKPSFKTVWQAVKYGLNG 120

A27 LENEVVNTVVAQEVKRLKLDRSAEFKNALAKLRAEAKKSGDDKKPSFKTVWQAVKYGLNG 120

A3 LENEVVNTVVAQEVKRLKLDRSAEFKNALAKLRAEAKKSGDDKKPSFKTVWQAVKYGLNG 120

A18 LENEVVNTVVAQEVKRLKLDRSAEFKNALAKLRAEAKKSGDDKKPSFKTVWQAVKYGLNG 120

A2 LENEVVNTVVAQEVKRLKLDRSAEFKNALAKLRAEAKKSGDDKKPSFKTVWQAVKYGLNG 120

A12 LENEVVNTVVAQEVKRLKLDRSAEFKNALAKLRAEAKKSGDDKKPSFKTVWQAVKYGLNG 120

A1 LENEVVNTVVAQEVKRLKLDRSAEFKNALAKLRAEAKKSGDDKKPSFKTVWQAVKYGLNG 120

A208 LENEVVNTVVAQEVKRLKLDQSAEFKDTLAKLRAEAKKSGDDKKPSFKTLWSALEYELNG 120

A215 LENEVVNTVVAQEVKRLKLDQSAEFKDTLAKLRAEAKKSGDDKKPSFKTLWSALEYELNG 120

*::*:..******** ****:*****: *.***:**:************:*.:::* ***

A206 RAYALHIAKTQPVSEQDAKAAYDNISGFYKGTQEVQLGEILTDKEENAKKAVAGLKAKKG 180

A36 RAYALHIAKTQPVSEQDAKAAYDNISGFYKGTQEVQLGEILTDKEDNAKKAVAGLKAKKG 180

A39 RAYALHIAKTQPVSEQDAKAAYDNISGFYKGTQEVQLGEILTDKEDNAKKAVAGLKAKKG 180

A40 RAYALHIAKTQPVSEQDAKAAYDNIRGFYKGTQEVQLGEILTDKEDNAKKAVAGLKAKKG 180

A34 RAYALHIAKTQPVSEQDAKAAYDNIRGFYKGTQEVQLGEILTDKEDNAKKAVAGLKAKKG 180

A204 RAYALHIAKTQPVSEQDAKAAYDNISGFYKGTQEVQLGEVLTDKEDNAKKAVADLKAKKG 180

A181 EAYALHIAKTQPVSEQEVKAAYDNISGFYKGTQEVQLGEILTDKEENAKKAVADLRAKKG 180

A216 EAYALHIAKTQPVSEQEVKAAYDNISGFYKGTQEVQLGEILTDKEENAKKAVADLKAKKG 180

A211 EAYALHIAKTQPVSEQEVKAAYDNISGFYKGTQEVQLGEILTDKEENAKKAVADLRAKKG 180

A197 EAYALHIAKTQPVSEQEVKAAYDNISGFYKGTQEVQLGEILTDKEENAKKAVADLKAKKG 180

A144 EAYALHIAKTQPVSEQEVKAAYDNISGFYKGTQEVQLGEILTDKEENAKKAVADLKAKKG 180

A140 EAYALHIAKTQPVSEQEVKAAYDNISGFYKGTQEVQLGEILTDKEENAKKAVADLKAKKG 180

A139 EAYALHIAKTQPVSEQEVKAAYDNISGFYKGTQEVQLGEILTDKEENAKKAVADLKAKKG 180

A191 EAYALHIAKTQPVSEQEVKAAYDNISGLYKGTQEVQLGEILTDKEENAKKAVADLKAKKG 180

A189 EAYALHIAKTQPVSEQEVKAAYDNISGFYKGTQEVQLGEILTDKEENAKKAVADLKAKKG 180

A130 EAYALHIAKTQPVSEQEVKAAYDNISGFYKGTQEVQLGEILTDKEENAKKAVADLKAKKG 180

A177 EAYALHIAKTQPVSEQEVKAAYDNINGFYKGTQEVQLGEILTDKEENAKKAVADLKAKKG 180

A106 EAYALHIAKTQPVSEQEVKAAYDNISGFYKGTQEVQLGEILTDKEENAKKAVADLKAKKG 180

A168 EAYALHIAKTQPVSEQEVKAAYDNISGFYKGTQEVQLGEILTDKEENAKKAVADLKAKKG 180

A162 EAYALHIAKTQPVSEQEVKAAYDNISGFYKGTQEVQLGEILTDKEENAKKAVADLKAKKG 180

A161 EAYALHIAKTQPVSEQEVKAAYDNISGFYKGTQEVQLGEILTDKEENAKKAVADLKAKKG 180

A160 EAYALHIAKTQPVSEQEVKAAYDNISGFYKGTQEVQLGEILTDKEENAKKAVADLKAKKG 180

A123 EAYALHIAKTQPVSEQEVKAAYDNISGFYKGTQEVQLGEILTDKEGNAKKAVADLKAKKG 180

A107 EAYALHIAKTQPVSEQEVKAAYDNISGFYKGTQEVQLGEILTDKEENAKKAVADLKAKKG 180

A78 EAYALHIAKTQPVSEQEVKAAYDNISGFYKGTQEVQLGEILTDKEENAKKAVADLKAKKG 180

A117 EAYALHIAKTQPVSEQEVKAAYDNISGFYKGTQEVQLGEILTDKEENAKKAVADLKAKKG 180

A116 EAYALHIAKTQPVSEQEVKAAYDNISGFYKGTQEVQLGEILADKEENAKKAVADLKAKKG 180

A114 EAYALHIAKTQPVSEQEVKAAYDNISGFYKGTQEVQLGEILTDKEENAKKAVADLKAKKG 180

A108 EAYALHIAKTQPVSEQEVKAAYDNISGFYKGTQEVQLGEILTDKEENAKKAVADLKAKKG 180

A97 EAYALHIAKTQPVSEQEVKAAYDNISGFYKGTQEVQLGEILTDKEENAKKAVADLKAKKG 180

A61 EAYALHIAKTQPVSEQEVKAAYDNISGFYKGTQEVQLGEILTDKEENAKKAVADLKAKKG 180

A58 EAYALHIAKTQPVSEQEVKAAYDNISGFYKGTQEVQLGEILTDKEENAKKAVADLKAKKG 180

A57 EAYALHIAKTQPVSEQEVKAAYDNISGFYKGTQEVQLGEILTDKEENAKKAVADLKAKKG 180

A94 EAYALHIAKTQPVSEQEVKAAYDNISGFYKGTQEVQLGEILTDKEENAKKAVADLKAKKG 180

A92 EAYALHIAKTQPVSEQEVKAAYDNISGFYKGTQEVQLGEILTDKEENAKKAVADLKAKKG 180

A73 EAYALHIAKTQPVSEQEVKAAYDNISGFYKGTQEVQLGEILTDKEENAKKAVADLKAKKG 180

A81 EAYALHIAKTQPVSEQEVKAAYDNISGFYKGMQEVQLGEILTDKEENAKKAVADLKAKKG 180

A79 EAYALHIAKTQPVSEQEVKAAYDNISGFYKGTQEVQLGEILTDKEENAKKAVADLRVKKG 180

A155 EAYALHIAKTQPVSEQEVKAAYDNISGFYKGTQEVQLGEILTDKEENAKKAVADLKAKKG 180

A153 EAYALHIAKTQPVSEQEVKAAYDNISGFYKGTQEVQLGEILTDKEENAKKAVADLKAKKG 180

A52 EAYALHIAKTQPVSEQEVKAAYDNISGFYKGTQEVQLGEILTDKEENAKKAVADLKAKKG 180

A27 EAYALHIAKTQPVSEQEVKAAYDNISGFYKGTQEVQLGEILTDKEENAKKAVADLKAKKG 180

A3 EAYALHIAKTQPVSEQEVKAAYDNISGFYKGTQEVQLGEILTDKEENAKKAVADLRAKKG 180

A18 EAYALHIAKTQPVSEQEIKAAYDNISGFYKGTQEVQLGEILTDKEENAKKAVADLKAKKG 180

A2 EAYALHIAKTQPVSEQEVKAAYDNISGFYKGTQEVQLGEILTDKEENAKKAVADLKAKKG 180

A12 EAYALHIAKTQPVSEQEVKAAYDNISGFYKGTQEVQLGEILTDKEENAKKAVADLKAKKG 180

A1 EAYALHIAKTQPVSEQEVKAAYDNISGFYKGTQEVQLGEILTDKEENAKKAVADLKAKKG 180

A208 RAYALHIAKTQPVSEQEVKAVYDNISGFYKGTQEVQLGEILTDKEENAKKAVADLKAKKG 180

A215 RVYALHIAKTQPVSEQEVKAVYDNISGFYKGTQEVQLGEILTDKEENAKKAVADLKAKKG 180

..**************: **.**** *:*** *******:*:*** ******* *:.***

A206 FDAVLKQYSLNDHTKQTGKPDGYVPLKDLEQGVPPLYQAIKDLKKGEFTATPLKNGDFYG 240

A36 FDAVLKQYSLNDHTKQTGKPDGYVPLKDLEQGVPPLYQAVKDLKKGEFTATPLKNGDFYG 240

A39 FDAVLKQYSLNDHTKQTGKPDGYVPLKDLEQGVPPLYQAVKDLKKGEFTATPLKNGDFYG 240

A40 FDAVLKQYSLNDHTKQTGKPDGYVPLKDLEQGVPPLYQAIKDLKKGEFTATPLKNGDFYG 240

A34 FDAVLKQYSLNDHTKQTGKPDGYVPLKDLEQGVPPLYQAIKDLKKGEFTATPLKNGDFYG 240

A204 FDAVLKQYSLNDRAKQTGAPDEYVPLKNLEQDAVPLYQAVKDLKKGEFTAVPLKNGDFYG 240

A181 FDAVLKQYSLNDRTKQTGAPVGYVPLKDLEQGVPPLYQAIKDLKKGEFTATPLKNGDFYG 240

A216 FDAVLKQYSLNDRTKQTGAPVGYVPLKDLEQGVPPLYQAIKDLKKGEFTATPLKNGDFYG 240

A211 FDAVLKQYSLNDRTKQTGAPVGYVPLKDLEQGVPPLYQAIKDLKKGEFTATPLKNGDFYG 240

A197 FDAVLKQYSLNDRTKQTGAPVGYVPLKDLEQGVPPLYQAIKDLKKGEFTATPLKNGDFYG 240

A144 FDAVLKQYSLNDRTKQTGAPVGYVPLKDLEQGVPPLYQAIKDLKKGEFTATPLKNGDFYG 240

A140 FDAVLKQYSLNDRTKQTGAPVGYVPLKDLEQGVPPLYQAIKDLKKGEFTATPLKNGDFYG 240

A139 FDAVLKQYSLNDRTKQTGAPVGYVPLKDLEQGVPPLYRAIKDLKKGEFTATPLKNGDFYG 240

A191 FDAVLKQYSLNDRTKQTGAPVGYVPLKDLEQGVPPLYQAIKDLKKGEFTATPLKNGDFYG 240

A189 FDAVLKQYSLNDRTKQTGAPVRYVPLKDLEQGVPPLYQAIKDLKKGEFTATPLKNGDFYG 240

A130 FDAVLKQYSLNDRTKQTGAPVGYVPLKDLEQGVPPLYQAIKDLKKGEFTATPLKNGDFYG 240

A177 FDAVLKQYSLNDRTKQTGAPVGYVPLKDLEQGVPPLYQAIKDLKKGEFTATPLKNGDFYG 240

A106 FDAVLKQYSLNDRTKQTGAPVGYVPLKDLEQGVPPLYQAIKDLKKGEFTATPLKNGDFYG 240

A168 FDAVLKQYSLNDRTKQTGAPVGYVPLKDLEQGVPPLYQAIKDLKKGEFTATPLKNGDFYG 240

A162 FDAVLKQYSLNDRTKQTGAPVGYVPLKDLEQGVPPLYQSIKDLKKGEFTATPLKNGDFYG 240

A161 FDAVLKQYSLNDRTKQTGAPVGYVPLKDLEQGVPPLYQAIKDLKKGEFTATPLKNGDFYG 240

A160 FDAVLKQYSLNDRTKQTGAPVGYVPLKDLEQGVPPLYQAIKDLKKGEFTATPLKNGDFYG 240

A123 FDAVLKQYSLNDRTKQTGAPVGYVPLKDLEQGVPPLYQAIKDLKKGEFTATPLKNGDFYG 240

A107 FDAVLKQYSLNDRTKQTGAPVGYVPLKDLEQGVPPLYQAIKDLKKGEFTATPLKNGDFYG 240

A78 FDAVLKQYSLNDRTKQTGAPVGYVPLKDLEQGVPPLYQAIKDLKKGEFTATPLKNGDFYG 240

A117 FDAVLKQYSLNDRTKQTGAPVGYVPLKDLEQGVPPLYQAIKDLKKGEFTATPLKNGDFYG 240

A116 FDAVLKQYSLNDRTKQTGAPVGYVPLKDLEQGVPPLYQAIKDLKKGEFTATPLKNGDFYG 240

A114 FDAVLKQYSLNDRTKQTGAPVGYVPLKDLEQGVPPLYQAIKDLKKGEFTATPLKNGDFYG 240

A108 FDAVLKQYSLNDRTKQTGAPVGYVPLKDLEQGVPPLYQAIKDLKKGEFTATPLKNGDFYG 240

A97 FDAVLKQYSLNDRTKQTGAPVGYVPLKDLEQGVPPLYQAIKDLKKGEFTATPLKNGDFYG 240

A61 FDAVLKQYSLNDRTKQTGAPVGYVLLKDLEQGVPPLYQAIKDLKKGEFTATPLKNGDFYG 240

A58 FDAVLKQYSLNDRTKQTGAPVGYVPLKDLEQGVPLLYQAIKDLKKGEFTATPLKNGDFYG 240

A57 FDAVLKQYSLNDRTKQTGAPVGYVPLKDLEQGVPPLYQAIKDLKKGEFTATPLKNGDFYG 240

A94 FDAVLKQYSLNDRTKQTGAPVGYVPLKDLEQGVPPLYQAIKDLKKGEFTATPLKNGDFYG 240

A92 FDAVLKQYSLNDRTKQTGAPVGYVPLKDLEQGVPPLYQAIKDLKKGEFTATPLKNGDFYG 240

A73 FDAVLKQYSLNDRTKQTGAPVGYVPLKDLEQGVPPLYQAIKDLKKGEFTATPLKNGDFYG 240

A81 FDAVLKQYSLNDRTKQTGAPVGYVPLKDLEQGVPPLYQAIKDLKKGEFTATPLKNGDFYG 240

A79 FDAVLKQYSLNDRTKQTGAPVGYVPLKDLEQGVPPLYQAIKDLKKGEFTATPLKNGDFYG 240

A155 FDAVLKQYSLNDRTKQTGAPVGYVPLKDLEQGVPPLYQAIKDLKKGEFTATPLKNGDFYG 240

A153 FDAVLKQYSLNDRTKQTGAPVGYVPLKDLEQGVPPLYQAIKDLKKGEFTATPLKNGDFYG 240

A52 FDAVLKQYSLNDRTKQTGAPVGYVPLKDLEQGVPPLYQAIKDLKKGEFTATPLKNGDFYG 240

A27 FDAVLKQYSLNDRTKQTGAPVGYVPLKDLEQGVPPLYQAIKDLKKGEFTATPLKNGDFYG 240

A3 FDAVLKQYSLNDRTKQTGAPVGYVPLKDLEQGVPPLYQAIKDLKKGEFTATPLKNGDFYG 240

A18 FDAVLKQYSLNDRTKQTGAPVGYVPLKDLEQGVPPLYQAIKDLKKGEFTATPLKNGDFYG 240

A2 FDAVLKQYSLNDRTKQTGAPVGYVPLKDLEQGVPPLYRAIKDLKKGEFTATPLKNGDFYG 240

A12 FDAVLKQYSLNDRTKQTGAPVGYVPLKDLEQGVPPLYQAIKDLKKSEFTATPLKNGDFYG 240

A1 FDAVLKQYSLNDRTKQTGAPVGYVPLKDLEQGVPPLYQAIKDLKKGEFTATPLKNGDFYG 240

A208 FDAVLKQYSLNDRTKQTGAPDGYVPLKDLEQGVPPLYQAIKDLKKGEFTATPLKNGDFYG 240

A215 FDAVLKQYSLNDHTKQTGAPVGYVPLKDLEQGVPPLYQAIKDLKKGEFTATPLKNGDFYG 240

************::**** * ** **:*** . **:::*****.****.*********

A206 VYYVNDRRDVKVPSFDEMKEQLTGDLQAERIDRAVGALLDKADIKPAE 288

A36 VYYVNDRRDVKVPSFDEMKEQLTGDLQAERIDRAVGALLDKADIKPAE 288

A39 VYYVNDRREVKVPSFDEMKEQLTGDLQAERIDRAVGALLDKADIKPAE 288

A40 VYYVNDRREVKVPSFDEMKEQLTGDLQAERIDRAVGALLDKADIKPAE 288

A34 VYYVNDRRDVKVPSFDEMKEQLTGDLQAERIDRAVGALLDKADIKPAE 288

A204 VYYVNDRRDVKVPSFDELKEQIAGDLQAERIDRAVGALLNKADIKPVK 288

A181 VYYVNDSREVKVPSFDEMKGQIAGNLQAERIDRAVGALLGKANIKPAK 288

A216 VYYVNDSREVKVPSFDEMKGQIAGNLQAERIDRAVGALLGKANIKPAK 288

A211 VYYVNDSREVKVPSFDEMKGQIAGNLQAERIDRAVGALLGKANIKPAK 288

A197 VYYVNDSREVKVPSFDEMKGQIAGNLQAERIDRAVGALLGKANIKPAK 288

A144 VYYVNDSREVKVPSFDEMKGQIAGNLQAERIDRAVGALLGKVNIKPAK 288

A140 VYYVNDSREVKVPSFDEMKGQIAGNLQAERIDRAVGALLGKANIKPAK 288

A139 VYYVNDSREVKVPSFDEMKGQIAGNLQAERIDRAVGALLGKANIKPAK 288

A191 VYYVNDSREVKVPSFDEMKGQIAGNLQAERIDRAVGALLGKANIKPAK 288

A189 VYYVNDSREVKVPSFDEMKGQIAGNLQAERIDRAVGALLGKANIKPAK 288

A130 VYYVNDSREVKVPSFDEMKGQIASNLQAERIDRAVGALLGKANIKPAK 288

A177 VYYVNDSREVKVPSFDEMKGQIAGNLQAERIDRAVGALLGKANIKPAK 288

A106 VYYVNDSREVKVPSFDEMKGQIAGNLQAERIDRAVGALLGKANIKPAK 288

A168 VYYVNDSREVKVPSFDEMKGQIAGNLQAERIDRAVGALLGKANIKPAK 288

A162 VYYVNDSREVKVPSFDEMKGQIAGNLQAERIDRAVGALLGKANIKPAK 288

A161 VYYVNDSREVKVPSFDKMKGQIAGNLQAERIDRAVGALLGKANIKPAK 288

A160 VYYVNDSREVKVPSFDEMKGQIAGNLQAERIDRVVGALLGKANIKPAK 288

A123 VYYVNDSREVKVPSFDEMKGQIAGNLQAERIDRAVGALLGKANIKPAK 288

A107 VYYVNDSREVKVPSFDERKGQIAGNLQAERIDRAVGALLGKANIKPAK 288

A78 VYYVNDSREVKVPSFDEMKGQIAGNLQAERIDRAVGALLGKANIKPAK 288

A117 VYYVNDSREVKVPSFDEMKGQIAGNLQAERIDRAVGALLGKANIKPAK 288

A116 VYYVNDSREVKVPSFDEMKGQIAGNLQAERIDRAVGALLGKANIKPAK 288

A114 VYYVNDSREVKVPSFDEMKGQIAGNLQAERIDRAVGALLGKANIKPAK 288

A108 VYYVNDSREVKVPSFDEMKGQIAGNLQAERIDRAVGALLGKANIKPAK 288

A97 VYYVNDSREVKVPSFDEMKGQIAGNLQVERIDRAVGALLGKANIKPAK 288

A61 VYYVNDSREVKVPSFDEMKGQIAGNLQAERIDRAVGALLGKANIKPAK 288

A58 VYYVNDSREVKVPSFDEMKGQIAGNLQAERIDRAVGALLGKANIKPAK 288

A57 VYYVNDSREVKVPSFDEMKGQIAGNLQAERIDRAVGALLGKANIKPAK 288

A94 VYYVNDSREVKVLSFDEMKGQIAGNLQAERIDRAVGALLGKANIKPAK 288

A92 VYYVNDSREVKVSSFDEMKGQIAGNLQAERIDRAVGALLGKANIKPAK 288

A73 VYYVNDSREVKVPSFDEMKGQIAGNLQAERIDRAVGALLGKANIKPAK 288

A81 VYYVNDSREVKVPSFDEMKGQIAGNLQAERIDRAVGALLGKANIKPAK 288

A79 VYYVNDSREVKVPSFDEMKGQIAGNLQAERIDRAVGALLGKANIKPAK 288

A155 VYYVNDSREVKVPSFDEMKGQIVGNLQAERIDRAVGALLGKANIKPAK 288

A153 VYYVNDSREVKVPSFDEMKGQIAGNLQAERIDRAVGVLLGKANIKPVK 288

A52 VYYVNDSREVKVPSFDEMKGQIAGNLQAERIDRAVGVLLGKANIKPAK 288

A27 VYYVNDSREVKVPSFDEMKGQIAGNLQAERIDRAVGALLGKANIKPAK 288

A3 VYYVNDSREVKVPSFDEMKGQIAGNLQAERIDRAVGALLGKANIKPAK 288

A18 VYYVNDSREVKVPSFDEMKGQIAGNLQAERIDRAVGALLGKANIKPAK 288

A2 VYYVNDSREVKVPSFDEMKGQIAGNLQAERIDRAVGALLGKANIKPAK 288

A12 VYYVNDSREVKVPSFDEMKGQIAGNLQAERIDRAVGALLGKANIKPAK 288

A1 VYYVNDSREVKVPSFDEMKGQIAGNLQAERIDRAVGALLGKANIKPAK 288

A208 VYYVNDRREVKVPSFDEMKGQIAGDLQAEQIDRAVGALLGKADIKPAK 288

A215 VYYVNDRRDVKVPSFDEMKGQIAGDLQAEQIDRAVGALLGKADIKPAK 288

****** *:*** ***: * *:..:**.*:***.**.** *.:***.:

**Humbert e*t al.* S2 Fig. Alignment of non-redundant NMB0345 (NEIS1825) amino acid sequences for *Neisseria meningitidis* isolates in the** [http://pubmlst‑org/perl/bigsdb/bigsdb‑pl?db=pubmlst_neisseria_isolates](http://pubmlst.org/perl/bigsdb/bigsdb.pl?db=pubmlst_neisseria_isolates)  **database and the additional 13 strains in our collection.** Database was accessed 01-03-2016.

Amino acid sequence alignments were generated using Clustal (<http://www.ebi.ac.uk/Tools/msa/clustalo/>) and a dendrogram was then assembled using the non-redundant sequences with Jalview 2.8 ([www.jalview.org](http://www.jalview.org)).

*Denotes identical amino acids; - denote position of amino acid change. A denotes Allele.
