# Supplementary material for: Vaccine Potential and Diversity of the Putative Cell Binding Factor (CBF, NMB0345/NEIS1825) Protein of Neisseria meningitidis
Source: PLoS One. 2016 Aug 9;11(8):e0160403. doi: 10.1371/journal.pone.0160403 (PMC4978444; doi:10.1371/journal.pone.0160403)
Supplement: S3 Fig — The numbers denote the average distance using % identity tree calculated by Jalview. A denotes Allele. (PPTX) [file pone.0160403.s003.pptx]

## Slide 1
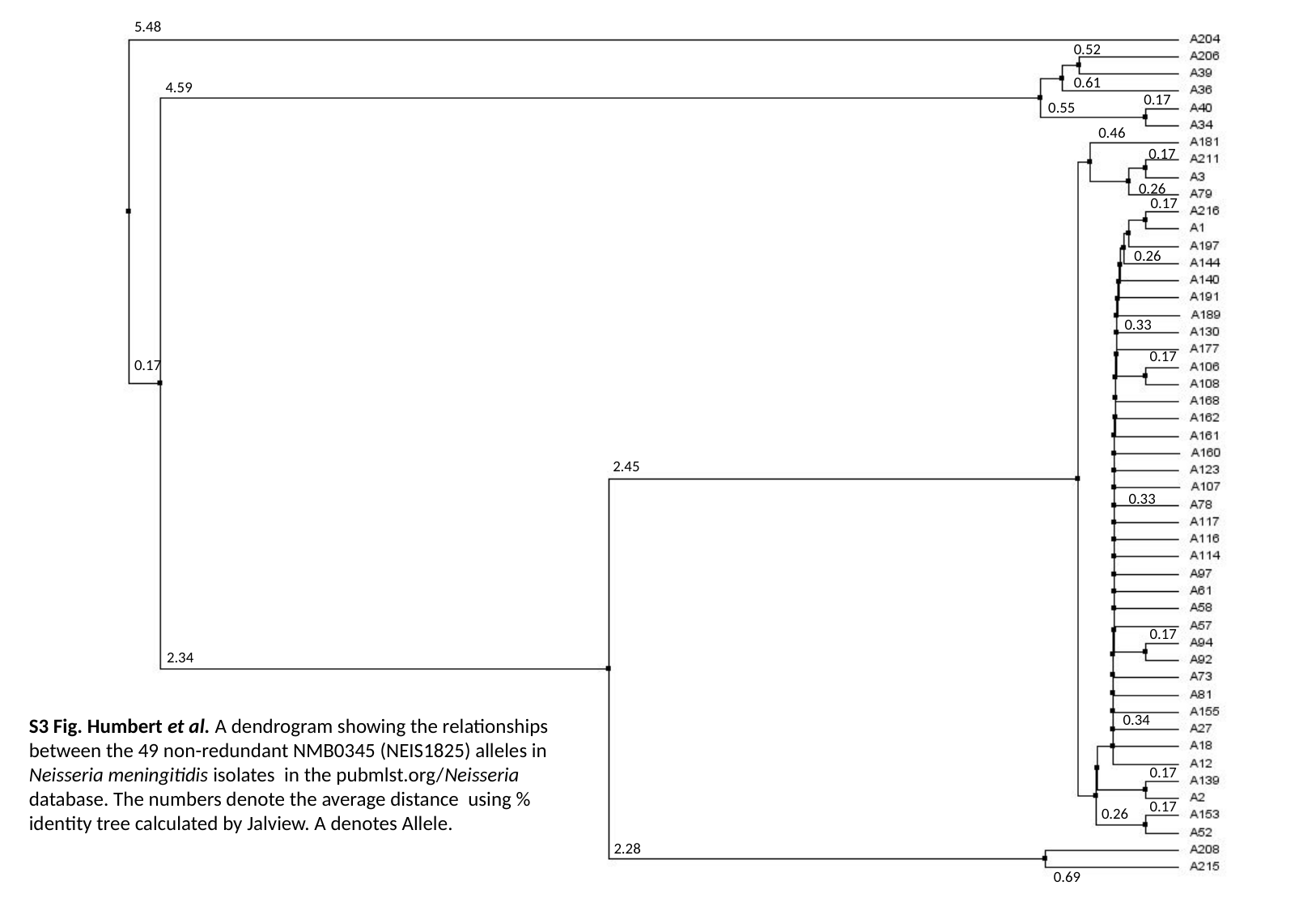

5.48
0.52
0.61
4.59
0.17
0.55
0.46
0.17
0.26
0.17
0.26
0.33
0.17
0.17
2.45
0.33
0.17
2.34
0.34
S3 Fig. Humbert et al. A dendrogram showing the relationships between the 49 non-redundant NMB0345 (NEIS1825) alleles in Neisseria meningitidis isolates in the pubmlst.org/Neisseria database. The numbers denote the average distance using % identity tree calculated by Jalview. A denotes Allele.
0.17
0.17
0.26
2.28
0.69
