# Supplementary material for: Vaccine Potential and Diversity of the Putative Cell Binding Factor (CBF, NMB0345/NEIS1825) Protein of Neisseria meningitidis
Source: PLoS One. 2016 Aug 9;11(8):e0160403. doi: 10.1371/journal.pone.0160403 (PMC4978444; doi:10.1371/journal.pone.0160403)
Supplement: S1 Table — Numbers in parentheses indicate that the Alleles produce proteins with identical amino acid sequences. Database was accessed 01-03-2106 and there are 136 allelic loci with isolates generating 49 non-redundant protein amino acid sequences. NG, no serogroup identified; ND, not determined. Table sorted numerically according to Alleles containing similar allelic proteins and then single Alleles. (DOCX) [file pone.0160403.s005.docx]

| **ALLELE NEIS1825 (NMB0345)** | **A** | **B** | **C** | **E** | **H** | **W** | **X** | **Y** | **Z** | **NG** | **ND** | **TOTAL** |
| --- | --- | --- | --- | --- | --- | --- | --- | --- | --- | --- | --- | --- |
| **1** (+4+5+6+7+8+11+13+14+17+19+23+24+25+45+48+49+53 +54+56+59+60+70+72+74+75+77+82+83+84+86+87+88+89+90+91+93+95+98+103+111+112+113+121+122+133+137+138+152+154+156+157+158+159+163+170+171+172+173+184+186+187+188+190+196+201+212+213+217) | 226 | 1833 | 431 | 20 | 1 | 913 | 29 | 728 | 10 | 166 | 619 | **4976** |
| **2** (+69+76+85+96+115+136+185+203) |  | 213 | 45 |  |  | 3 | 1 |  | 1 | 19 | 21 | **303** |
| **3** (+132+135) |  | 72 | 493 |  |  | 4 |  |  |  | 7 | 23 | **599** |
| **18** (+71+80+99+100+109+169) |  | 541 | 3 |  |  | 1 | 2 |  |  | 8 | 33 | **588** |
| **81**(+214) |  |  | 1 |  |  |  |  | 29 |  |  | 6 | **36** |
| **94** (+134) |  | 1 |  |  |  | 2 |  |  |  |  |  | **3** |
| **97** (+110) |  | 2 |  |  |  |  |  |  |  |  | 3 | **5** |
| **12** | 4 |  |  |  |  |  |  |  |  |  |  | **4** |
| **27** |  |  |  |  |  |  |  | 11 |  | 2 | 2 | **15** |
| **34** |  |  |  |  |  |  |  |  |  |  | 2 | **2** |
| **36** |  |  |  |  |  |  |  |  |  |  | 1 | **1** |
| **39** |  | 1 |  |  |  |  |  |  |  |  | 1 | **2** |
| **40** |  |  |  |  |  |  |  |  |  | 1 |  | **1** |
| **52** |  | 10 | 1 | 1 |  |  |  | 5 | 2 | 25 | 1 | **45** |
| **57** |  | 2 | 7 |  |  | 1 |  |  |  |  |  | **10** |
| **58** |  | 1 |  |  |  |  |  |  |  |  |  | **1** |
| **61** |  |  |  |  |  |  |  | 3 |  |  |  | **3** |
| **73** |  | 1 |  |  |  |  |  |  |  |  |  | **1** |
| **78** |  |  |  |  |  |  |  | 2 |  |  |  | **2** |
| **79** |  | 1 | 9 |  |  |  |  |  |  |  |  | **10** |
| **92** |  | 1 |  |  |  |  |  |  |  |  |  | **1** |
| **106** |  |  |  |  |  |  |  | 1 |  |  |  | **1** |
| **107** |  |  |  |  |  | 1 |  |  |  |  |  | **1** |
| **108** |  |  |  |  |  |  |  | 1 |  |  |  | **1** |
| **114** |  | 3 |  |  |  |  |  |  |  |  |  | **3** |
| **116** |  | 1 |  |  |  |  |  |  |  |  |  | **1** |
| **117** |  |  |  |  |  |  |  | 1 |  |  |  | **1** |
| **123** |  |  |  |  |  |  |  |  |  |  | 1 | **1** |
| **130** |  |  |  |  |  |  |  |  |  | 1 |  | **1** |
| **139** |  | 1 |  |  |  |  |  |  |  |  |  | **1** |
| **140** |  | 1 |  |  |  |  |  |  |  |  |  | **1** |
| **ALLELE NEIS1825 (NMB0345)** | **A** | **B** | **C** | **E** | **H** | **W** | **X** | **Y** | **Z** | **NG** | **ND** |  |
| **144** |  | 2 |  |  |  |  |  |  |  |  |  | **2** |
| **153** |  |  |  |  |  |  |  | 1 |  |  |  | **1** |
| **155** |  |  | 1 |  |  |  |  |  |  |  |  | **1** |
| **160** |  |  |  |  |  |  |  | 1 |  |  |  | **1** |
| **161** |  |  |  |  |  | 1 |  |  |  |  |  | **1** |
| **162** |  |  |  |  |  |  |  |  | 1 |  |  | **1** |
| **168** |  | 1 |  |  |  |  |  |  |  |  |  | **1** |
| **177** |  | 1 |  |  |  |  |  |  |  |  |  | **1** |
| **181** |  | 1 |  |  |  |  |  |  |  |  |  | **1** |
| **189** |  | 1 |  |  |  |  |  |  |  |  |  | **1** |
| **191** |  |  |  |  |  |  |  |  |  |  | 1 | **1** |
| **197** |  |  |  |  |  |  |  |  |  | 1 |  | **1** |
| **204** |  |  |  |  |  |  |  |  |  | 1 |  | **1** |
| **206** |  |  |  |  |  |  |  |  |  |  | 1 | **1** |
| **208** |  |  |  |  |  |  |  |  |  |  | 1 | **1** |
| **211** |  | 1 | 4 |  |  |  |  |  |  |  |  | **5** |
| **215** |  |  |  |  |  |  |  |  |  | 1 |  | **1** |
| **216** |  |  |  |  |  |  |  | 2 |  |  |  | **2** |
| **TOTAL** | **230** | **2692** | **995** | **21** | **1** | **926** | **32** | **785** | **14** | **232** | **716** | **6644** |

**S1 Table Humbert *et al.*: Analysis of NMB0345 (NEIS1825) alleles and number of isolates per serogroup:** data are collated from <http://pubmlst.org/perl/bigsdb/bigsdb.pl?db=pubmlst_neisseria_isolates> and also include the 13 strains from our collection. Numbers in parentheses indicate that the Alleles produce proteins with identical amino acid sequences. Database was accessed 01-03-2106 and there are 136 allelic loci with isolates generating 49 non-redundant protein amino acid sequences. NG, no serogroup identified; ND, not determined. Table sorted numerically according to Alleles containing similar allelic proteins and then single Alleles.
