# Supplementary material for: Vaccine Potential and Diversity of the Putative Cell Binding Factor (CBF, NMB0345/NEIS1825) Protein of Neisseria meningitidis
Source: PLoS One. 2016 Aug 9;11(8):e0160403. doi: 10.1371/journal.pone.0160403 (PMC4978444; doi:10.1371/journal.pone.0160403)
Supplement: S2 Table — Numbers in parentheses indicate that the Alleles produce proteins with identical amino acid sequences. Database was accessed 01-03-2016 for the UK from 2013–2015, the most recent data. NG, no serogroup identified; ND, not determined. (DOCX) [file pone.0160403.s006.docx]

| **ALLELE NEIS1825 (NMB0345)** | **A** | **B** | **C** | **E** | **H** | **W** | **X** | **Y** | **Z** | **NG** | **ND** | **Other** | **Total** |
| --- | --- | --- | --- | --- | --- | --- | --- | --- | --- | --- | --- | --- | --- |
| **1** (+4+5+7+11+13+14+17+19+45+53 +54+56+70+72+74+77+82+86+87 +111+112+113+170+171+172+184  +186+187+188+201) |  | 554 | 38 |  |  | 381 |  | 221 |  | 11 | 7 |  | **1212** |
| **2** (+69+115+185+203) |  | 52 | 1 |  |  |  |  |  |  |  |  |  | **53** |
| **3** |  | 6 | 37 |  |  |  |  |  |  |  |  |  | **43** |
| **18** (+71) |  | 158 | 1 |  |  | 1 |  |  |  | 3 | 1 |  | **164** |
| **27** |  |  |  |  |  |  |  | 1 |  | 1 |  |  | **2** |
| **39** |  | 1 |  |  |  |  |  |  |  |  |  |  | **1** |
| **52** |  | 1 |  |  |  |  |  |  |  |  |  |  | **1** |
| **81** |  |  | 1 |  |  |  |  | 17 |  |  |  |  | **18** |
| **94** |  |  |  |  |  | 1 |  |  |  |  |  |  | **1** |
| **110** |  | 1 |  |  |  |  |  |  |  |  |  |  | **1** |
| **114** |  | 3 |  |  |  |  |  |  |  |  |  |  | **3** |
| **116** |  | 1 |  |  |  |  |  |  |  |  |  |  | **1** |
| **117** |  |  |  |  |  |  |  | 1 |  |  |  |  | **1** |
| **189** |  | 1 |  |  |  |  |  |  |  |  |  |  | **1** |
| **204** |  |  |  |  |  |  |  |  |  | 1 |  |  | **1** |
| **Unassigned** |  | 16 |  | 1 |  |  | 1 | 6 |  | 9 |  | 1 | **34** |
| **Total** |  | 794 | 78 | 1 | 0 | 383 | 1 | 246 | 0 | 25 | 8 | 1 | **1537** |

**S2 Table Humbert *et al.*: Analysis of NMB0345 (NEIS1825) alleles and number of isolates per serogroup for UK data 2013-2015:** data are collated from <http://pubmlst.org/perl/bigsdb/bigsdb.pl?db=pubmlst_neisseria_isolates>. Numbers in parentheses indicate that the Alleles produce proteins with identical amino acid sequences. Database was accessed 01-03-2016 for the UK from 2013-2015, the most recent data. NG, no serogroup identified; ND, not determined.
